# Opposite Effects of Added AsPh_3_ Reveal a Drastic Mechanistic Switch in Rh^I^/Au^I^ Transmetalations via Rh–Au Bonded Intermediates

**DOI:** 10.1021/acs.inorgchem.5c01081

**Published:** 2025-06-26

**Authors:** Marconi N. Peñas-Defrutos, Camino Bartolomé, Max García-Melchor, Pablo Espinet

**Affiliations:** † IU CINQUIMA/Química Inorgánica, Facultad de Ciencias, 16782 Universidad de Valladolid, 47071 Valladolid, Spain; ‡ School of Chemistry, CRANN and AMBER Research Centres, Trinity College Dublin, College Green, D02 PN40 Dublin 2, Ireland; § Center for Cooperative Research on Alternative Energy (CIC EnergiGUNE), Basque Research and Technology Alliance (BRTA), 01510 Vitoria-Gasteiz, Spain; ∥ IKERBASQUE, Basque Foundation for Science, 48009 Bilbao, Spain

## Abstract

In contrast with the previously reported decelerating effect of added L (L = AsPh_3_) on the Rf/Pf exchange reaction between [Au­(Pf)­L] and *trans*-[Rh­(Rf)­(CO)­L_2_] (Pf = C_6_F_5_; Rf = C_6_F_3_Cl_2_-3,5), the Rf/Cl exchange between [AuClL] and *trans*-[Rh­(Rf)­(CO)­L_2_] is accelerated by addition of an excess of L. By combining experimental data and microkinetic modeling, with DFT calculations, the unexpected existence of two cooperative Rf/Cl exchange mechanisms is demonstrated. The opposite kinetic effects of L addition, from negative in the Rf/Pf exchange process, opposing L dissociation in an octahedral rhodium intermediate, to positive in the Rf/Cl exchange, opening an L-catalyzed alternative pathway via tricoordinate gold intermediates, explain the Janus effect of AsPh_3_. The three transmetalation pathways involve a metal redox-insertion step with accessible activation barrier, producing intermediates with Rh–Au bonds. Whereas our previously reported Rf/Pf exchange implied Rh­(I) oxidation by Au­(I), the Rf/Cl exchange mechanism involves Au­(I) oxidation by Rh­(I). Further support is provided by NBO studies, which reveal remarkable electronic donations from the oxidized metal in each case forging the M–M′ covalent interaction in the intermediates yielded by the redox-insertion step.

## Graphical abstract

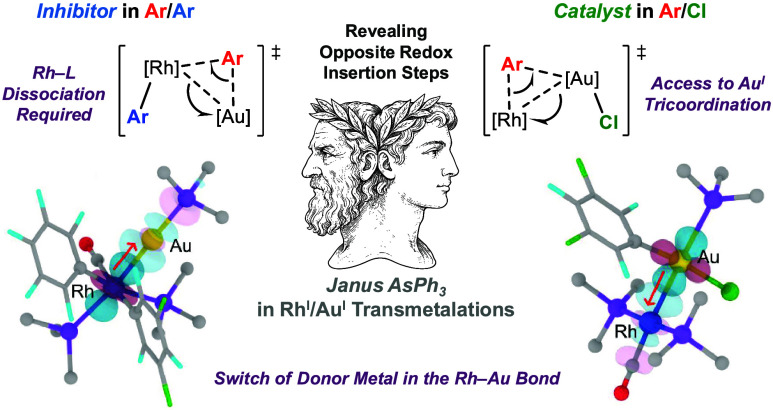

## Introduction

A most common transmetalation in catalysis is R for X exchange (R = organic group on a main group element; X = halide on a transition metal). It usually occurs via μ-X μ-R double-bridged intermediates. For other transmetalations, such as R/R′ exchanges (responsible for undesired homocoupling products), different mechanisms have been reported. For instance, while for the ClAu^I^/PhSn^IV^ exchange, the classical double-bridged mechanism was reported, for the (vinyl)­Au^I^/PhSn^IV^ transmetalation, an oxidative addition/reductive elimination (OA/RE) pathway, via an intermediate featuring an Au–Sn bond, was found.^1^

In the growing field of bimetallic catalysis, which deals with homogeneous processes where either two transition metals (TM),^2^ or one TM and one group-11 element,^3^ cooperate in a catalytic transformation (often a C–C coupling), the exchange mechanisms can follow diverse pathways. Examples are the Pd/Au cooperativity in gold cocatalyzed Stille coupling of bulky aryls,^4^ or the now popular Pd–Cu catalyzed cross-coupling processes.^5^ Better understanding of the transmetalation mechanisms operating in these processes may facilitate the design of more efficient systems.

The scarcity of mechanistic studies on Rh^I^/Au^I^ transmetalations prompted us to investigate this promising catalytic dyad.^6,7^ In this context, we have already reported the mechanism of aryl/aryl’ scrambling between the Vaska-type complex *trans*-[Rh­(Rf)­(CO)­(AsPh_3_)_2_] (**1**) (Rf = C_6_F_3_Cl_2_-3,5) and [Au­(Pf)­(AsPh_3_)] (Pf = C_6_F_5_), shown in eq 1.^8^ The unconventional fractional and negative rate dependence of this aryl/aryl’ exchange upon free AsPh_3_ addition (Figure 1A) suggested a rather complex reaction mechanism. In fact, this exchange follows an OA/RE pathway via redox insertion of Rh into the Au–C bond), which indicates that Rh^I^ is formally oxidized by Au^I^. Figure 1B depicts the computed transition state illustrating the insertion. This initial step leads to an intermediate with octahedral Rh, linear Au, and a Rh^II^–Au^0^ bond.^9^ The M/M′ redox process looks quite reasonable considering the oxidizing power of Au­(I). The retarding effect of added AsPh_3_ is due to a rate determining isomerization step in the transmetalation pathway, which requires AsPh_3_ dissociation from Rh in the octahedral intermediate resulting from the redox insertion.^8^

1

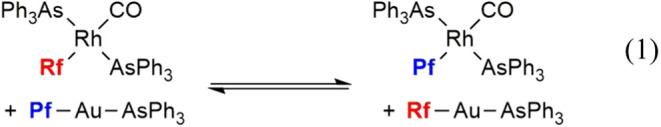




**Figure 1:**
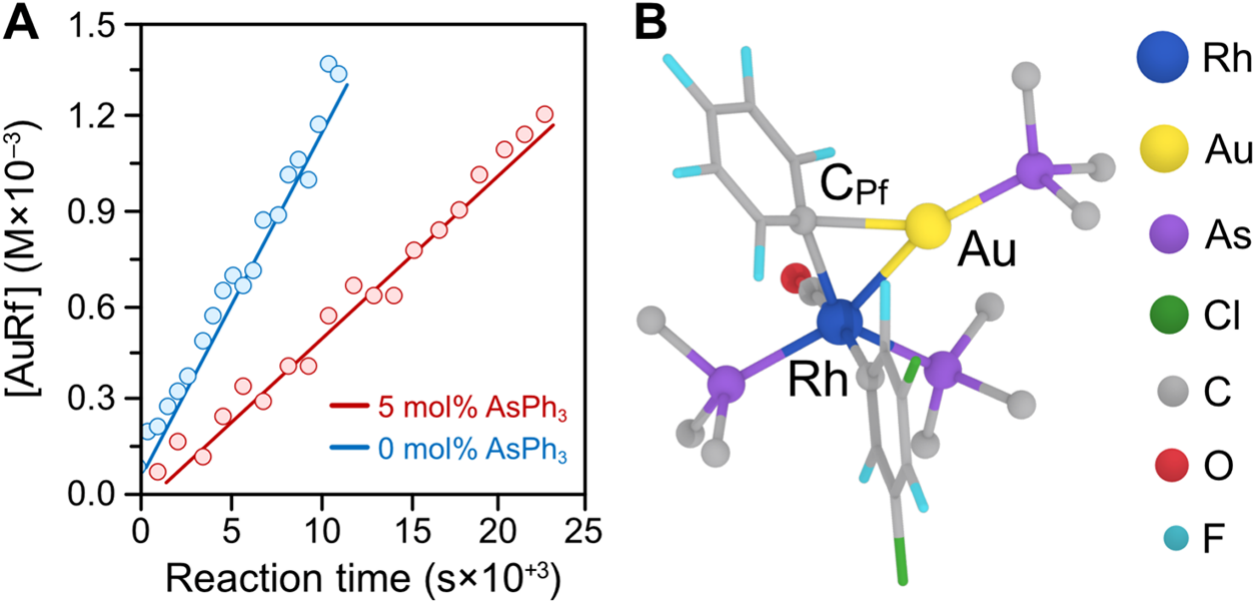
(A) Kinetic effect of added ligand on the transmetalation rate in eq 1. (B) DFT-calculated transition state for the Rh insertion into the Au–C_Pf_ bond. The Ph groups in the arsine are simplified to their C_ipso_ for clarity. Figure adapted with permission from ref 8. Copyright [2019]­[Wiley].

## Results and Discussion

### Experimental Kinetic Studies

Herein we report the Rf/Cl transmetalation reaction between *trans*-[Rh­(Rf)­(CO)­(AsPh_3_)_2_] (**1**) and [AuCl­(AsPh_3_)] (**2**) yielding *trans*-[RhCl­(CO)­(AsPh_3_)_2_] (**3**) + [Au­(Rf)­(AsPh_3_)] (**4**) (eq 2).^10^ The use of Cl in the gold reagent, instead of Pf, seems to produce a dramatic mechanistic change hinted by the fact that the addition of AsPh_3_ accelerates the process instead of retarding it.^8^ More precisely, AsPh_3_ has a catalytic effect on the Rf/Cl exchange. 
2

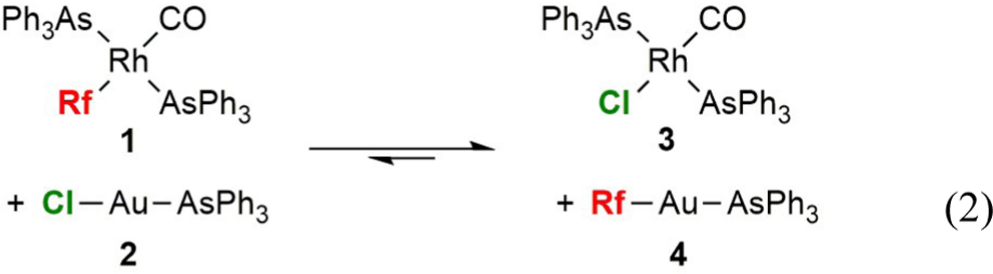

 In CD_2_Cl_2_, the exchange equilibrium of (eq 2) is largely shifted to formation of **3** + **4**. It was monitored by ^19^F NMR in both senses, using stoichiometric conditions (1:1 molar ratio) until steady concentrations of the species were reached (24 h at room temperature).^11^ This afforded an equilibrium constant *K*_eq_ = 7.0 × 10^3^ (Δ*G*_0_ = −5.2 kcal mol^–1^) corresponding to 99% conversion to **3** + **4** (Figure S1), revealing complex **1** as a thermodynamically efficient aryl transmetalating agent to gold­(I). Neither other products nor reaction intermediates were detected. Experiments using 2:1 or 1:2 Au:Rh ratios doubled the reaction rate observed for the stoichiometric 1:1 reaction, confirming first order reaction kinetics in both reactants (Figure S2). Finally, as mentioned above, the addition of AsPh_3_ resulted in a significant and unexpected increase of reaction rate, in striking contrast with the clear deceleration shown in the case of Rf/Pf exchange.^8^

Figure 2 plots the experimental points of the ^19^F NMR monitoring of the exchanges with different additions of free AsPh_3_ (CD_2_Cl_2_, 273 K). Least squares adjustment of the data for the reaction in the absence of added AsPh_3_ (Figure 2, red dots) yielded an initial reaction rate *r*_0_ = 2.16 × 10^–7^ mol L^–1^ s^–1^, corresponding to a Gibbs activation energy Δ*G*_273 K_^‡^ = 19.2 kcal mol^–1^ (see kinetic section in the SI for details). The addition of only 10 mol % AsPh_3_ relative to gold concentration accelerated the initial transmetalation rate by *ca*. 60% (deep blue dots), and a 4-fold rate increase was observed with 60 mol % of AsPh_3_ (pink dots). Hence, the observed AsPh_3_ dependence is not linear. Moreover, the transmetalation rate seems to level off at large overstoichiometric ligand concentrations and subsequent additions of AsPh_3_ to the last sequence of points represented (500% mol of AsPh_3_, light blue dots) hardly produce any significant rate change (see Table S2 for details).

**Figure 2:**
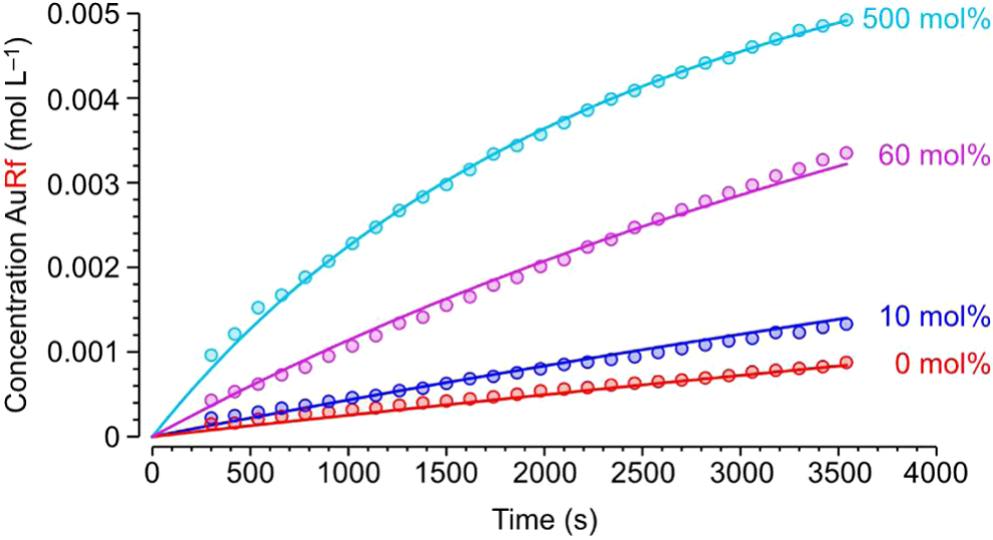
Concentration vs time data (circles) obtained by monitoring the formation of product [Au­(Rf)­(AsPh_3_)] (**4**) by means of ^19^F NMR in CD_2_Cl_2_ at 273 K with different percentages of added AsPh_3_. Initial concentrations of the reactants: [Rh]_0_ = [Au]_0_ = 1.0 × 10^–2^ mol L^–1^. Lines represent data adjusted with COPASI software.

The continuous lines in Figure 2 correspond to the overall adjustment of all the data, using COPASI software,^12^ to the kinetic model shown in Scheme 1. The model consists of two competitive pathways (**A** and **B**) connected by an equilibrium of AsPh_3_ coordination to **2**, forming the tricoordinate species [AuCl­(AsPh_3_)_2_] (**5**). The reaction without added AsPh_3_ proceeds through pathway **A**, whereas in the presence of added AsPh_3_ the two pathways sum up. **A** and **B** give the same end products, hence pathway **B** is an AsPh_3_-catalyzed variation of pathway **A**.

**Scheme 1:**
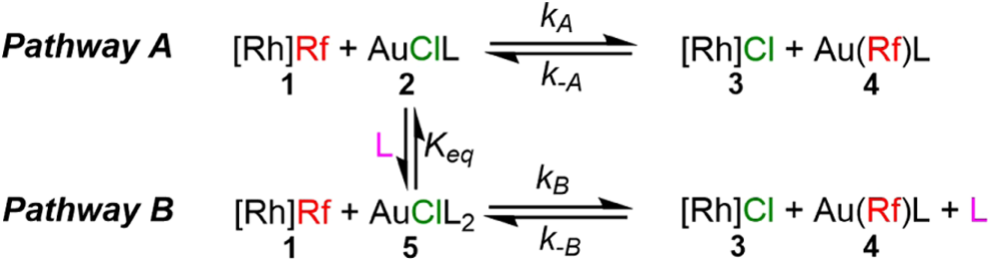
Kinetic Model for COPASI Fitting of the AsPh_3_ Catalytic Effect. L = AsPh_3_. Ligands in the Rh Complex Omitted for Clarity

This mechanistic proposal is based on a study reporting that expansion of the Au­(I) linear coordination of [AuX­(AsPh_3_)] complexes occurs upon AsPh_3_ addition, giving rise to multiple coordination equilibria involving [AuX­(AsPh_3_)_*m*_] structures (X = halide; *m* = 1, 2, 3). These structures display progressive elongation of the Au–X bond upon successive coordinations of AsPh_3_.^13^ In our study, intermediate **5**, lacking ^19^F, cannot be detected in the ^19^F NMR monitoring but mass spectrometry confirmed the formation of [AuCl­(AsPh_3_)_2_] (**5**) in CH_2_Cl_2_ with substoichiometric amounts of AsPh_3_, whereas [AuCl­(AsPh_3_)_3_], plausible to be formed at large excess of AsPh_3_, was not detected. On the other hand, the ^19^F NMR spectra detected only [Au­(Rf)­(AsPh_3_)] (**4**) but not [Au­(Rf)­(AsPh_3_)_2_]. This is consistent with the fact that the gold center in **4** is more electron rich than in **5**, hence less electrophilic and less prone to tricoordination.^14^

The satisfactory COPASI fitting to the experimental points in Figure 2 strongly supports the proposed mechanisms and affords a value of *K*_*eq*_ = 2.14 × 10^2^ mol^–1^ L (Δ*G*_eq_ = – 2.9 kcal mol^–1^) for the fast AsPh_3_ coordination equilibrium leading to formation of **5**. Moreover, the activation energy barrier obtained independently by least-squares adjustment of the reaction without added AsPh_3_ (pathway **A**) is virtually identical to the value obtained in the overall COPASI fitting: Δ*G*_**A**_^‡^ = 19.2 kcal mol^–1^. The formation of **5**, induced by the addition of free AsPh_3_, opens a faster transmetalation pathway **B**, with Δ*G*_**B**_^‡^ = 17.8 kcal mol^–1^ (Scheme 1).

This work combines experimental and computational approaches to elucidate the cooperative Rf/Cl exchange pathways (**A** and **B**) using density functional theory (DFT) at the wb97xd level (see SI for details).^15,16^ The kinetic experiments define the energetic profile of the process—including the relative positions of reactants and products, the rate-determining transition states, and the thermodynamics of AsPh_3_ coordination—with high precision. In contrast, structural information on intermediates and transition states, which cannot be accessed experimentally, is provided by DFT calculations, which have been satisfactorily benchmarked against the available experimental data. To reflect this complementary use of both approaches, Figures 3 and 10 depict the Gibbs energy diagrams using a two-color scheme: black for computed values and blue for experimental data, which are used as reference due to their lower uncertainty.^17^

### Computational Study of Transmetalation Pathway **A**

The Gibbs energy diagram of pathway **A** is shown in Figure 3. The first step involves the interaction of complexes **1** and **2** to yield a weakly interacting van der Waals complex (***I1***), which lies 3.8 kcal mol^–1^ above the energy of the separated reactants. From this intermediate, the redox-insertion of the Au center into the Rh–C bond takes place via ***TS1***, featuring a fairly symmetric Rf bridge, with a COPASI-refined activation energy of 19.2 kcal mol^–1^.^18^ This step results in formation of ***I2***, where both metal centers display square planar geometry. Using the carbene insertion processes as reaction model, the two electrons of the new Au–Rh bond in ***I2*** have been provided by gold (gold has been oxidized) while Rh has received this donation and consequently has been reduced. This finding confirms the suspected mechanistic switch from Rh^I^ redox-insertion, found in our previous study,^8^ to Au^I^ redox-insertion here. Considering that the electronegativity differences in M–M′ bonds are small, the accepted rules to assign formal metal-oxidation numbers state that M–M′ bonds do not count (as applied in metals themselves, where the structures have many M–M bonds but the formal oxidation state is assigned M^0^). Consequently, we should consider ***I2*** closer to a Au^II^–Rh^0^ bimetallic molecule than to a Au^III^–Rh^–I^ bonded species,^9^ suggesting a milder oxidation (e.g., 1e donation) of the initial Au^I^ compound (**2**) and milder reduction (e.g., 1e acceptance) of the Rh^I^ reagent (**1**).^19^

**Figure 3:**
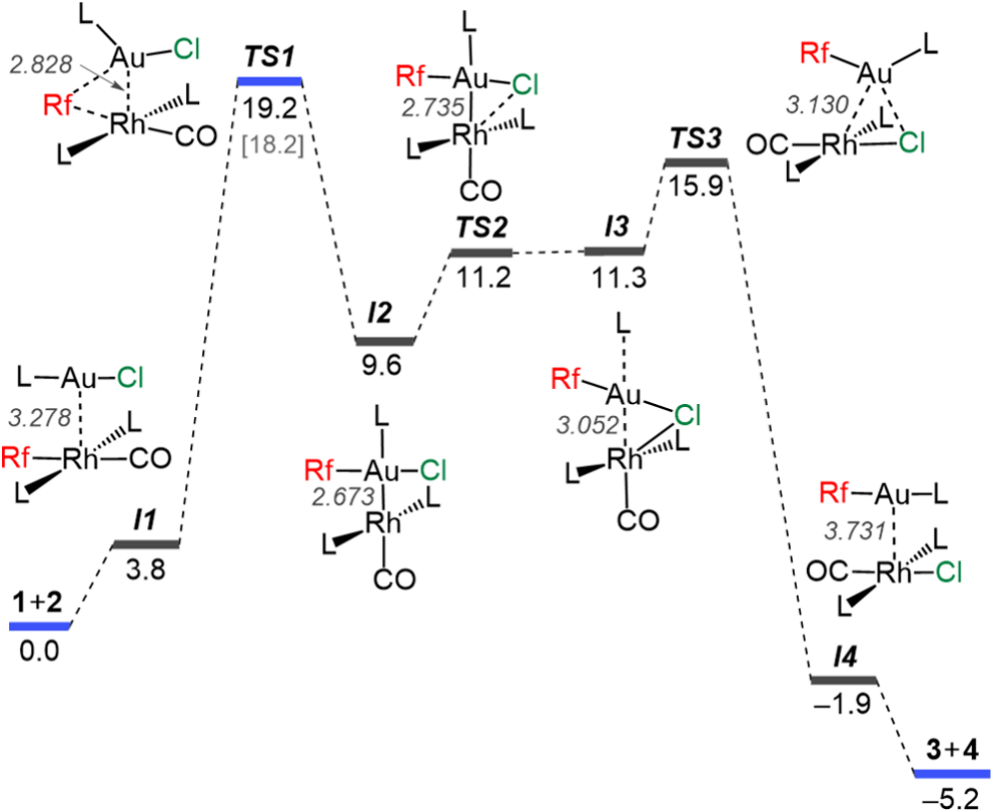
Gibbs energy diagram (in kcal mol^–1^) for the transmetalation reaction between **1** and **2** in CH_2_Cl_2_ via pathway **A**. Rh–Au distances (Å) are also given. Blue lines highlight COPASI-refined values obtained from the experimental data. The DFT-calculated energy for ***TS1*** is shown in brackets for comparison.

Detailed analysis of the optimized structures of ***TS1*** and ***I2*** (Figure 4) shows that, while the Rf group acts as a bridging group between both metals in ***TS1*** (with typical M–C_ipso_ distances of *ca*. 2.3 Å), the Cl^–^ ligand remains coordinated to gold and behaves as a mere spectator. Hence, the formation of a Rf bridge and a strong Au–Rh interaction (2.828 Å in ***TS1***) is preferred over transmetalation via potential Rf/Cl mixed double bridges, which would presumably involve weaker M–M′ interactions. Indeed, all our attempts to find such a double-bridged transition state with a feasible activation energy were unsuccessful. Consistently, the Au–Rh bond distance in intermediate ***I2*** (2.673 Å), somewhat shorter than the sum of covalent radii (2.78 Å),^20^ is comparable to those found in single-crystal X-ray diffraction structures of related complexes (2.531 and 2.690 Å).^21^ Interestingly, the remarkably long Au–As bond observed in ***I2*** is indicative of a large *trans influence* of the Au–Rh bond.^22^

**Figure 4:**
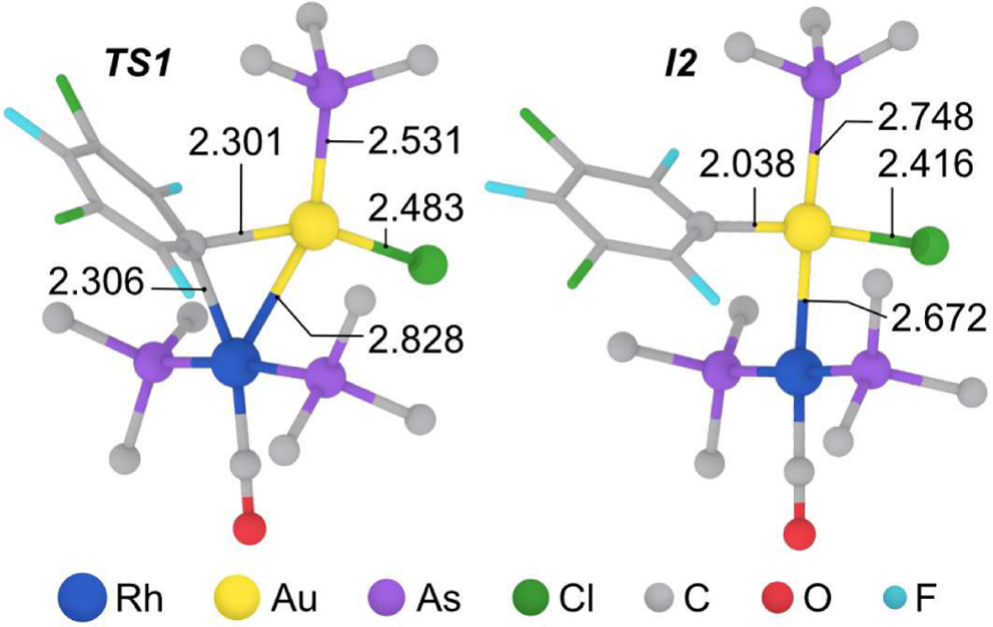
Ball and stick representation of the optimized structures of ***TS1*** (left) and ***I2*** (right) with selected bond distances (in Å). Ph groups in the AsPh_3_ ligands are omitted for clarity. **Sum of covalent radii (in Å)**: Au–Rh = 2.78; Au–C = 2.09; Rh–C = 2.15; Au–As = 2.55; Au–Cl = 2.38.^20^

In the next steps, transmetalation of the Cl group occurs in a process involving two transition states (***TS2*** and ***TS3***) of relatively low energy. In particular, ***TS2*** features an incipient very asymmetric Au–Cl–Rh bridge with a long Rh–Cl distance, and a relative energy barrier from ***I2*** of 1.6 kcal mol^–1^. In contrast, intermediate ***I3*** displays a fairly symmetric Au–Cl–Rh bridge with Au–Cl and Rh–Cl distances moderately longer than the sum of covalent radii. The Cl transfer from Au in ***I3*** to Rh in ***I4*** is completed via ***TS3***. In this transfer the gold atom recovers the electron density involved in the Au–Rh bond, generating the van der Waals complex ***I4*** with the reaction products weakly interacting.

### Does Rh­(I) Oxidize Au­(I)?

The results of our previous study on the aryl exchange between [Au­(Pf)­(AsPh_3_)] (Pf = C_6_F_5_) and *trans*-[Rh­(Rf)­(CO)­(AsPh_3_)_2_] (Rf = C_6_F_3_Cl_2_-3,5) (**1**) had shown that the process started with an oxidative insertion of the rhodium­(I) center into the Au–C_6_F_5_ bond, which, as discussed, led to the formation of the Rh­(II)–Au(0) intermediate [(AsPh_3_)_2_(CO)­(Rf)­(Pf)­Rh–Au­(AsPh_3_)] (***I2^∧^***) (Figure 5, left).^8^ Herein, we have found that the Rf/Cl exchange reaction between *trans*-[Rh­(Rf)­(CO)­(AsPh_3_)_2_] (**1**) and [AuCl­(AsPh_3_)] (**2**) is triggered by an oxidative insertion of the Au­(I) center into the Rh–Rf bond, which corresponds electronically to a less expected oxidation of the Au^I^ center by Rh^I^, yielding the Rh(0)–Au­(II) intermediate [(AsPh_3_)_2_(CO)­Rh–Au­(Rf)­(Cl)­(AsPh_3_)] (***I2***) (Figure 5, right).

**Figure 5:**
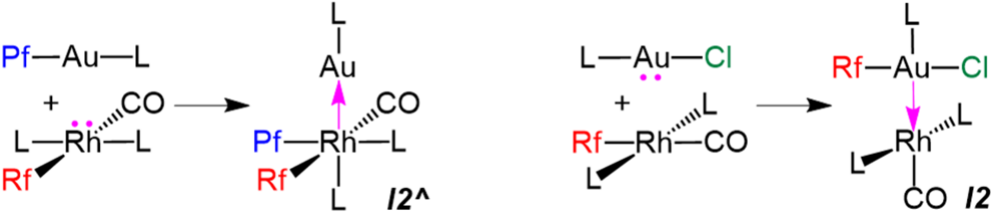
Reagents vs key intermediates for reactions in eqs 1 (left) and 2 (right). The donor electron pair and the dative covalent M→M′ bond formed in each case are highlighted in pink.

The joint analysis of the two redox-insertion mechanisms for the Rh/Au transmetalations, Rf/Cl here and Rf/Pf in our previous study, gives us the opportunity to consider chemical concepts operating in bimetallic species with M–M′ bonds. First of all, the idea behind the rule that metal–metal bonds do not count in the assignment of formal oxidation states of metal centers is that in highly polar bonds involving atoms of very different electronegativity (*e.g*. Rh–Rf or Au–Cl) the two bond electrons are assigned to the more electronegative group ([Rh^+^]–[:Rf^–^] or [Au^+^]–[:Cl^–^] to reflect the loss of electron density. Thus, these bonds count as +1 in the formal oxidation state of the Rh or Au centers. The electronic polarization in metal–metal bonds (*e*.*g*. Rh–Au) is much smaller, and the bond electron pair is oversimplified by the classical rule, which considers this pair as equally shared by the two metal centers (zero polarization). Obviously, this is true only when the metal–metal bond connects two identical [M] fragments. Moreover, speaking of bond electron pairs is also an oversimplification because the orbitals involved in the M–M′ bond are neither purely metal orbitals nor fully centered at the metal and consequently, the potentially bonding orbitals are deeply influenced by the ligands. Anyhow, this extreme but useful approximation to bond polarization and electron density at the metal center allows us to assign Rh^I^ and Au^I^ oxidation states to complexes such as **1**–**5**. These traditional concepts can benefit from sophisticated theoretical approximations. In this line, some recent reports deal with Sanderson’s principle^23^ of ″electronegativity *equalization*″ during bond formation,^24,25^ or with the concept of ″chemical potentials equalization″, leading to ″electronegativity *equilibration*″.^26^ Here we use different computational approaches to compare the two transmetalation mechanisms found.

### QTAIM (Quantum Theory of Atoms in Molecules) Study

The two exchange mechanisms discussed so far have in common the occurrence of metal redox-insertions with comparable DFT activation energies (***TS1*** = 18.2 kcal mol^–1^; ***TS1^∧^*** = 20.3 kcal mol^–1^),^27^ which give rise to the strikingly different Rh–Au bonded intermediates depicted in Figure 5, namely ***I2*** (Au and Rh are square planar) and ***I2^∧^*** (octahedral Rh and linear Au) highlighting the mechanistic divergence between Rf/Cl and Rf/Pf transmetalations. Although the Au–Rh bond does not count for the oxidation state number, it obviously counts for the coordination number.

Figure 6 shows the electron density maps of ***I2*** (left) and ***I2** **^∧^*** (right), obtained by means of QTAIM calculations (details in SI),^28^ allowing to easily identify a bond critical point (BCP 3, −1) for each Rh–Au bond. The calculated electron density at the Rh–Au BCP is lower for ***I2*** than for ***I2^∧^*** (ρ = 5.33 × 10^–2^ and 6.85 × 10^–2^ respectively), as could be expected for a longer Rh–Au bond distance in ***I2*** (Rh–Au = 2.672 Å in ***I2***; Rh–Au = 2.602 Å in ***I2^∧^***; sum of covalent radii = 2.78 Å).^20^ Interestingly, the direct comparison of the M–BCP distances, and the opposite variations observed for each metal in both cases (see caption of Figure 6), further supports the changes in the Rh–Au bond polarizations exaggerated in Figure 5 as donations of an electron pair.

**Figure 6:**
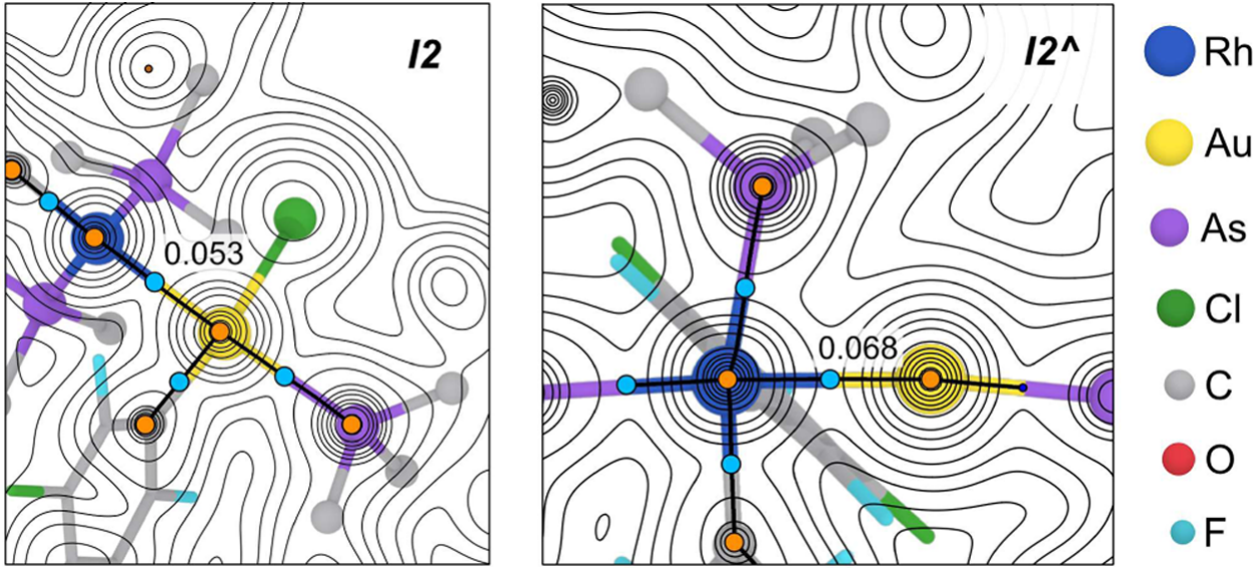
Topological maps obtained by QTAIM for ***I2*** (left) and ***I2^∧^*** (right), including the electron densities (ρ­(r)) found at the Rh–Au BCP. Blue and orange dots denote bond and nuclear critical points respectively. Relevant distances in Å: ***I2***: Rh–Au = 2.672; Rh–BCP = 1.231; Au–BCP = 1.441. ***I2^∧^***: Rh–Au = 2.602; Rh–BCP = 1.316; Au–BCP = 1.286. Experimental covalent radii in Å: Rh = 1.42(7); Au = 1.36(6).^20^

Extending the conventional use of sum of covalent radii to predict bond distances, we could formally consider the **Rh–BCP** and **BCP–Au** distances in each intermediate as their effective metallic radii (*r*_ef_). The *r*_ef_ for Rh is larger in ***I2^∧^*** (1.316 Å), where Rh is supposed to have been oxidized, polarizing some bond electron density to the domains of Au, than in ***I2*** (1.231 Å). Conversely, the *r*_ef_ for Au is larger in ***I2*** (1.442 Å), in which the gold center has presumably polarized some bond electron density in favor of Rh, than in ***I2^∧^*** (1.286 Å). Thus, the *r*_ef_ elongation is somehow reflecting, in each case, the direction of electron polarization of the Rh–Au covalent bond pair. We propose that a larger *r*_ef_ reflects a higher polarization of the electron density of the corresponding metal center (equivalent to ″oxidation″) toward the counterpart in the M–M′, which undergoes ″reduction″ with concomitant shrinking of its *r*_ef_. The QTAIM results support the *a priori* ″intuitive″ Rh^I^ ″oxidation″ with Rh→Au electron pair polarization in ***I2^∧^***, and the ″counterintuitive″ Au^I^ oxidation, by Au→Rh electron pair polarization, in ***I2*** (Figure 5).

### NBO (Natural Bonding Orbital) and SOPT (Second Order Perturbation Theory) Analyses

We are aware that the previous description of the M–M′ bond in ***I2*** and ***I2^∧^***, although useful, is somehow naive since the Rh···Au interactions cannot be assigned only to a bond electron pair and the orbitals involved are not purely metal orbitals.^24^ Further insight on the nature of these interactions can be obtained with NBO and SOPT analyses on the redox insertion transition states (***TS1*** and ***TS1^∧^***) and on the key intermediates (***I2*** and ***I2^∧^***) of both mechanisms. This allows us to identify the main donor–acceptor interactions involving participation of metal orbitals in each case (Figures 7 and 8). The ″oxidations″, ″reductions″ and bond polarizations proposed in the previous sections are well supported by the NBO results (Table 1).

**Table 1:** Selected Donor–Acceptor Interactions Identified by Means of NBO Analyses on ***TS1***/***TS1*** ^***∧***^ and on ***I2***/***I2*** ^***∧***^ Illustrated in Figures 7 and 8, Respectively^a^

**species**	**donor**	**contribution**	**acceptor**	**contribution**	***E***_**SOPT**_
***TS1***	BD Au–C_Rf_	88% C s (28%) p (72%)	BD* Rh–C_CO_	73% Rh s (46%) d (54%)	100.4
		12% Au s (95%) p (1%) d (4%)		27% C s (65%) p (35%)	
***TS1^∧^***	BD Rh–C_Pf_	83% C s (28%) p (72%)	LV Au	Au s (93%) d (7%)	129.3
		17% Rh s (56%) d (44%)			
***I2***	LP Au	Au s (7%) d (93%)	BD* Rh–C_CO_	73% Rh s (46%) d (54%)	33.8
				27% C s (65%) p (35%)	
***I2^∧^***	LP Rh	Rh s (1%) d (99%)	BD* Au–As	77% Au s (92%) d (8%)	45.0
				23% As s (33%) p (67%)	

a *LP*, *LV*, *BD* and BD* stand for Lone Pair, Low Valence, Bonding and Antibonding orbitals respectively. SOPT energies in kcal mol^–1^.

**Figure 7:**
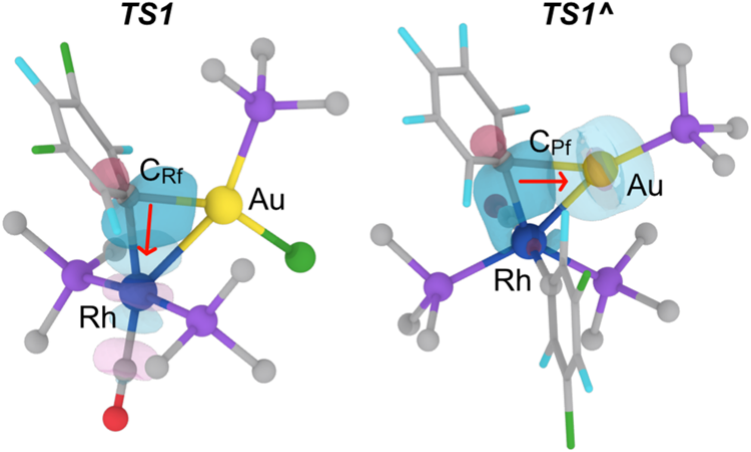
Simplified representation of ***TS1*** (left) and ***TS1^∧^*** (right), with isosurfaces (isovalue = 0.07 au) of selected NBOs involved in the transmetalation. The red arrows indicate the sense of electron donation. Ph groups in the AsPh_3_ ligands are omitted for clarity. F: light blue; Cl: green. As: violet; O: red.

**Figure 8:**
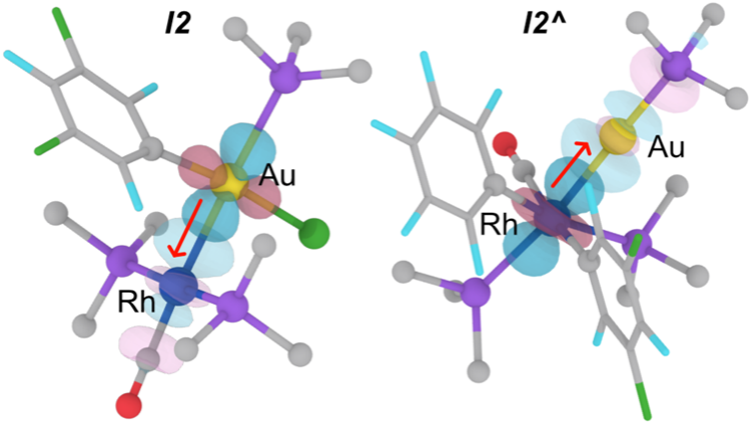
Isosurfaces (isovalue = 0.07 au) of selected NBOs. Left: Au^II^→Rh^0^ intermediate (***I2***) in Rf/Cl exchange. Right: Rh^II^→Au^0^ intermediate (***I2^∧^***) in Rf/Pf exchange. Ph groups in the AsPh_3_ ligands are omitted for clarity.

The dissimilar behavior of the two gold complexes [Au­(X)­(AsPh_3_)] (X = C_6_F_5_, Cl) is related with the preferred orthogonal approximations of the linear gold molecules to the Vaska-type *trans*-[Rh­(Rf)­(CO)­(AsPh_3_)_2_] (**1**) square-planar complex, shown in Figures 5 and 7. In the transition state ***TS1*** the linear gold molecule [AuCl­(AsPh_3_)] approaches the Rh center from above the Rf–Rh–CO axis where the Rh center is electron poorer (***TS1*** in Figure 7) and concerted slippage leads to insertion of the electron pair of the Au atom into the Rf–Rh bond. The donor orbital in ***TS1*** is the bonding orbital of the incipient Au–Rf bond, with the electron density coming mostly from C_ipso_ (88% C, 28% s and 72% p character), and only 12% from Au (95% s character). The acceptor is a hybrid Rh–CO antibonding orbital (BD*), with 73% Rh contribution (46% s and 54% d character) and 27% C contribution (65% s and 35% p character). Overall, for the metals this interaction entails a partial Au oxidation by Rh with an associated SOPT energy of 100.4 kcal mol^–1^ (Table 1).

In contrast, the linear complex [Au­(C_6_F_5_)­(AsPh_3_)] approximates to the same rhodium reactant **1** over its (Ph_3_As)–Rh–(AsPh_3_) axis where the Rh center is electron richer (***TS1^∧^*** in Figure 7),^8^ and concerted slippage occurs with bending of the (Ph_3_As)–Rh–(AsPh_3_) angle, leading to insertion of the Rh atom into the Au–Pf bond. The donor orbital in ***TS1^∧^*** is a bonding orbital (BD) from the incipient Rh–Pf bond, mainly centered at the C_ipso_ of the bridging aryl (83% C_Pf_, with 28% s and 72% p character; 17% Rh, with 56% s and 44% d character). The acceptor is a low valence (LV) Au orbital constituted mostly by a gold s atomic orbital (93%). This interaction implies a partial oxidation of Rh by Au with an associated SOPT energy of 129.3 kcal mol^–1^.

Activation strain analyses (details in SI) illustrate that the higher interaction energy between reactive fragments (*E*_int_) observed for ***TS1^∧^*** compared to ***TS1*** (71.3 vs 64.5 kcal mol^–1^) is compensated by the also higher negative contribution of the sum of distortion energies (*E*_dist_) for ***TS1^∧^*** (see Table S5). This accounts for their similar activation barriers.

Geometry relaxations from both transition states, either evolution from ***TS1*** to ***I2*** or from ***TS1^∧^*** to ***I2^∧^***, complete the metal insertion processes. The dissimilar polarizations of the electron density in the Au­(μ-C_Ar_)Rh bridge in each case (Figure 7), lead to formation of the Rh–Au bonds in ***I2*** or ***I2^∧^*** with opposite donor and acceptor metal centers (Figure 8).

Figures 5 and 8 depict the main NBO interactions identified in the respective Rh–Au bonds of intermediates ***I2*** and ***I2^∧^***. For ***I2***, we can speak of a Au^II^→Rh^0^ donation where a filled Au *sd* orbital (93% d) acts as donor, and the acceptor is an antibonding Rh–CO orbital (73% Rh, 27% C_CO_) mainly centered at the Rh atom (*E*_SOPT_ = 33.8 kcal mol^–1^). Presumably, the CO ligand makes rhodium a better acceptor, allowing for the formal oxidation of the gold center. Conversely, a Rh^II^→Au^0^ donation is found for ***I2^∧^***, where the donor is an occupied lone-pair Rh orbital (99% *d character)*, and the acceptor is an antibonding Au–As orbital (77% Au, 23% As) mainly centered at the Au atom (*E*_SOPT_ = 45.0 kcal mol^–1^). Notably, no interactions with comparable SOPT energies are found for donations from the corresponding reduced metals (see Table S4 for more NBO data).

In summary, NBO and SOPT analyses certainly support that the Au–Rh bond is polarized toward Rh in ***I2*** whereas the opposite holds true for ***I2^∧^***. Consequently, Au^I^ is in fact ″oxidized″ by Rh^I^, or more precisely by Rh^I^(CO), in the transmetalation reaction of *trans*-[Rh­(Rf)­(CO)­(AsPh_3_)_2_] with [AuCl­(AsPh_3_)], whereas it is reduced by Rh^I^ in the analogous reaction with [Au­(C_6_F_5_)­(AsPh_3_)]. However, this overall balance cannot be correctly understood if the effects of other atoms in the molecule are disregarded.

It should be noted that the analysis on which metal center undergoes the equivalent to partial oxidation or partial reduction along the mechanistic pathway (at the transition states and intermediates) should not be confused with the net result of the exchange (eqs 1 and 2) since no formal change in oxidation state occurs between reactants and products in either case.^29,30^

### Electronic Structure Evidence for Rhodium Reduction in Intermediate ***I2***

To further support the Rh(0)–Au­(II) formulation proposed for intermediate ***I2***, a localized orbital analysis was carried out using the Pipek–Mezey method.^31^ Careful examination of the molecular orbitals (MOs) of the molecule allowed the identification of the four occupied d-type valence orbitals centered on the rhodium atom. The resulting localized orbitals closely match the canonical d_*xy*_, d_*xz*_, d_*yz*_, and d_z^2^_ functions and account for 8 d-electrons (Figure 9). The topology and relative energies of the localized orbitals are also consistent with a distorted square-planar geometry, with d_*xz*_ and d_*yz*_ orbitals clearly nondegenerate due to the lack of D_4h_ symmetry.^32^

**Figure 9:**
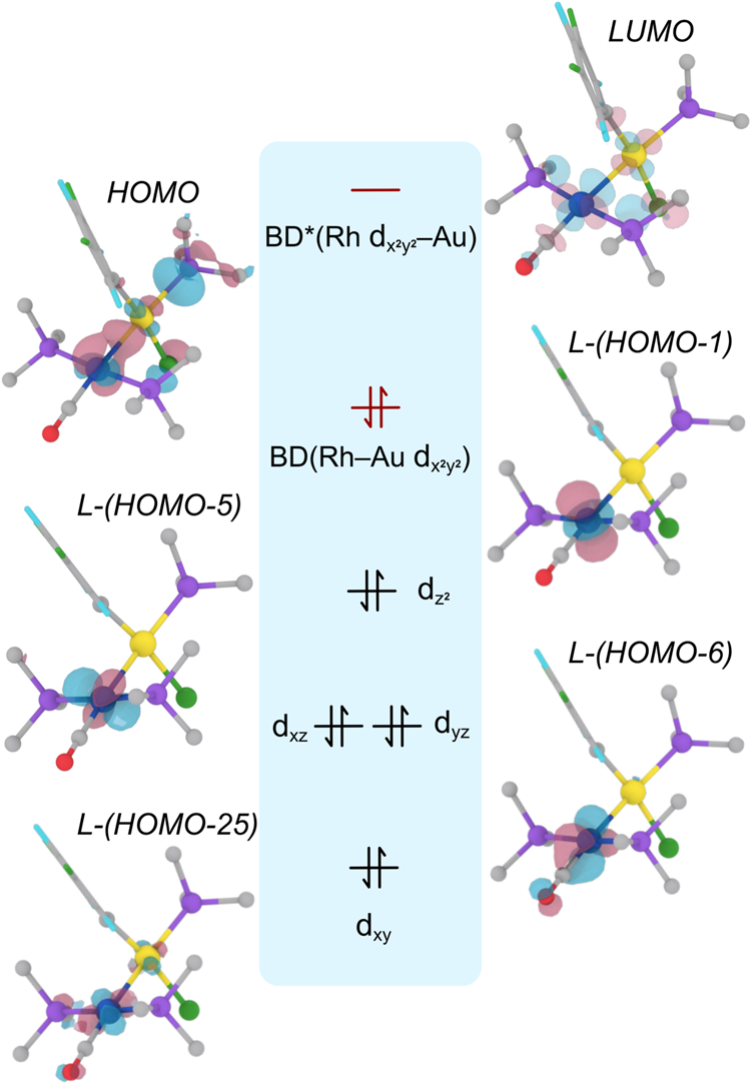
Pipek–Mezey localized rhodium d-orbitals (d_*xy*_, d_*xz*_, d_*yz*_, and d_z^2^_), HOMO, and LUMO of intermediate ***I2***. Ph groups in the AsPh_3_ ligands are omitted for clarity. Idealized orbital distribution for a D_4h_ complex with d^8^ configuration is included as comparison, highlighting the inclusion of the shared Rh–Au HOMO.

In addition, the HOMO, though not localized, unambiguously places d-type electron density on rhodium, primarily originating from gold. This orbital forms the bonding component along the Rh–Au axis and is consistent with a dative Au→Rh interaction. Conversely, the LUMO displays the corresponding Rh–Au antibonding counterpart, involving the rhodium d_x^2^–y^2^_ atomic orbital (Figure 9). These results reinforce the formal d^9^ configuration for rhodium in ***I2***, implying a Rh(0) oxidation state, and more importantly, confirm the unexpected ability of gold­(I) to reduce rhodium­(I).

For intermediate ***I2^∧^***, featuring an octahedral rhodium geometry, localization of the Rh-centered d-orbitals was not feasible due to extensive mixing with ligand-based orbitals. However, the HOMO and LUMO (Figure S6) clearly show the bonding and antibonding components along the Rh–Au axis, respectively, allowing identification of the rhodium d_z^2^_ orbital as the main contributor to the dative Rh→Au interaction. Although the unoccupied Rh d_x^2^–y^2^_ orbital could not be directly localized, its contributions are evident in LUMO+3 and LUMO+5. These findings further support the formal oxidation of rhodium in ***I2^∧^***, consistent with a rhodium­(II) oxidation state.

### Computational Study of the Transmetalation Pathway **B**

The AsPh_3_-catalyzed character of the transmetalation pathway **B** suggests AsPh_3_ coordination to gold (Scheme 1) and, in fact, it involves exchange between 3-coordinate [AuCl­(AsPh_3_)_2_] (**5**) and *trans*-[Rh­(Rf)­(CO)­(AsPh_3_)_2_] (**1**). As commented before, mass spectra confirm the formation of **5** upon AsPh_3_ addition to [AuCl­(AsPh_3_)] (**2**) in CH_2_Cl_2_, and discard significant formation of [AuCl­(AsPh_3_)_3_] in the experimental conditions. The proposed Gibbs energy diagram for pathway **B** is depicted in Figure 10 and shows that the coordination of AsPh_3_ induces the formation of ionic transition states and intermediates with dissociated Cl^–^. Obviously, the thermodynamic balance of the catalyzed reaction is identical to the uncatalyzed one (Δ*G*_0_ = −5.2 kcal mol^–1^).

**Figure 10:**
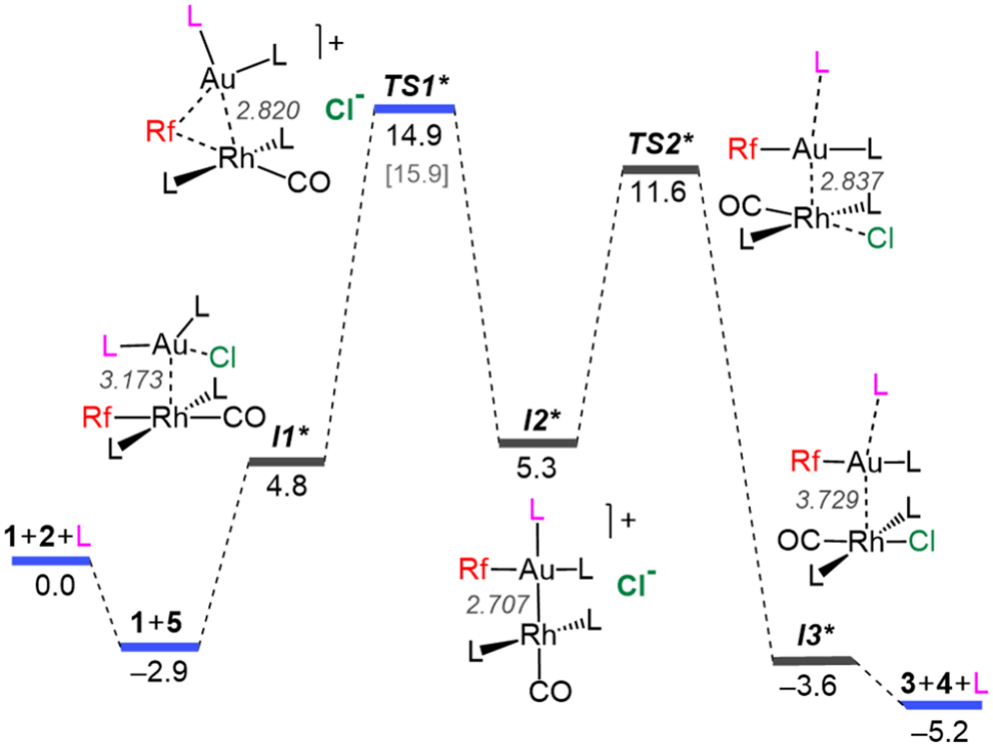
Gibbs energy diagram (in kcal mol^–1^) for the ligand-promoted transmetalation between **1** and **5** (Pathway **B**) in CH_2_Cl_2_. Rh–Au distances (in Å) are also given. Blue lines denote COPASI-refined values obtained from experimental data. The energy of I2* was referenced to the cationic TS1*. The DFT-calculated energy for TS1* is shown in brackets for comparison (note that the overall barrier should be evaluated from the lowest energy point).

The equilibrium constant for the formation of **1** + **5** obtained from the microkinetic simulation corresponds to a stabilization of Δ*G*_eq_ = −2.9 kcal mol^–1^ associated with gold tricoordination. The van der Waals intermediate I1* (added AsPh_3_ molecule highlighted in pink) shows already a substantially elongated Au–Cl bond while both AsPh_3_ ligands remain tightly coordinated to the gold center. Subsequently, the rate determining redox insertion occurs as in the uncatalyzed case. The tricoordination of gold in **5**, increasing its electron density compared to the linear [AuCl­(AsPh_3_)] (**2**), seems to facilitate notably this step, lowering the overall activation barrier to 17.8 kcal mol^–1^ for pathway **B**, compared to the Δ*G* ^‡^ for pathway **A** (19.2 kcal mol^–1^, Figure 3). In contrast with the otherwise analogous ***TS1***, the Au­(μ-Rf)Rh transition state TS1* (Rh–Au = 2.820 Å), features a dissociated Cl^–^ ligand, displaced from the Au coordination sphere by the additional AsPh_3_ ligand, forming an ion-pair. Similarly to Pathway A, the hypothetical concerted mechanism involving a Au­(μ-Rf)­(μ-Cl)Rh transition state was computationally discarded.

Relaxation from TS1* leads to I2*, which also displays an ion-pair nature. Structurally, the cation of I2* features the expected elongation of the Au–As bond trans to Rh, and the Au–Rh distance (2.707 Å) supports a covalent bond similar to the one in ***I2***. From I2*, Cl^–^ coordination to Rh induces, via TS2*, extrusion of the [AuRfL] (**4**) gold moiety from the Rh coordination sphere leading to I3* with concomitant dissociation of the L ligand confirming the catalytic role of arsine.

The participation of ionic species along a reaction mechanism often compromises the accuracy of computational results, benchmarked against the experimental values, due to non-negligible DFT errors intimately related with poor description of solvation effects using implicit solvent models.^33^ It is well-known that ionic molecules show only low conductivities in CH_2_Cl_2_, so it is reasonable to propose the formation of ion pairs upon AsPh_3_ addition, instead of separated ions. Contacted ion-pair structures, as proposed for TS1* and I2* in Figure 10, are expected for the AsPh_3_-promoted mechanism in CH_2_Cl_2_ because of its weak ion solvating strength. In favor of this proposal, when the reaction is carried out in acetone-d_6_ (a much better ion-solvating agent than CH_2_Cl_2_), the accelerating effect of added AsPh_3_ becomes remarkably higher.

The influence of the solvent on the efficiency of AsPh_3_ as a transmetalation catalyst is noteworthy. Specifically, while the addition of 10 mol % of free ligand increases the reaction rate by 60% in CD_2_Cl_2_ (red vs deep blue traces in Figure 2), the same AsPh_3_ loading in acetone-d_6_ results in a 3.1-fold acceleration compared to the uncatalyzed system (Figure S4), making the catalytic effect approximately five times greater. Unfortunately, a full kinetic analysis in this solvent was not feasible due to limited product solubility and extremely fast reaction rates at higher AsPh_3_ loadings. Nevertheless, these observations provide strong experimental support for the involvement of charged species in the catalyzed transmetalation mechanism. We hypothesize that the superior ability of acetone to solvate fully separated ions—unlike the more likely ion-pairing scenario in CD_2_Cl_2_—leads to a larger activation energy gap between the neutral (A) and ionic (B) pathways, resulting in a more pronounced catalytic effect.

The intimate details of ion-pair species in CD_2_Cl_2_ solutions are very complicated.^34^ Fortunately, the simple ion-pair species TS1* and I2* dealt with in the calculations seem to represent reasonably well the system in CH_2_Cl_2_ solution, since the computed activation energy from **1** + **5** (18.8 kcal mol^–1^) is remarkably close to the experimental COPASI-fitted value (17.8 kcal mol^–1^). In addition, the structures of the isolated cations of TS1* and I2* were optimized (details in SI) and were found to show only negligible differences with the cationic fragments of their respective ion pairs species confirming that, in the rate limiting aryl transfer, the chloride plays a spectator role in the structural rearrangement at the cation. Finally, it is worth mentioning that both pathways, either the uncatalyzed (**A**) or the AsPh_3_-catalyzed (**B**), unveiled for the Rf/Cl exchange (Scheme 1), have in common the formal oxidation of the gold center in the redox insertion step, as supported by the Au→Rh interactions found by the NBO studies on TS1* and I2* (see Figure S7).

## Conclusions

The joint analysis of the Rh^I^–Rf/Au^I^–Cl (Rf = C_6_F_3_Cl_2_-3,5) and Rh^I^–Rf/Au^I^–Pf (Pf = C_6_F_5_) transmetalation reactions reported here and in our previous paper,^8^ shows that they do not follow the prototypical mechanism involving transition states with mixed double bridges, but via redox-insertion of Au into the Rh–C_Rf_ bond, or of Rh into the Au–C_Pf_ bond, respectively. The preferred orthogonal approximation of X–Au–L (L = AsPh_3_) to the square planar *trans*-[RhRf­(CO)­L_2_] (**1**) (Cl–Au–L lying on the Rf–Rh–CO axis, or Pf–Au–L lying on the L–Rh–L axis) determines the redox insertion operating (Figure 5). This study should be taken as a warning to prevent careless mechanistic misinterpretations when combining two transition metal (or group 11) complexes and might find further application in the bimetallic catalysis field.

The reaction intermediates formed upon redox insertion contain Rh–Au bonds at distances shorter than the sum of the covalent radii. In the Rf/Pf exchange, ***I2^∧^*** featuring an octahedral Rh and linear Au was found, and further mechanistic evolution requires Rh–AsPh_3_ dissociation previous to the rate-determining isomerization step, explaining the decelerating effect upon arsine addition reported in ref 8. In contrast, the Rf/Cl exchange implies Au insertion into the Rh–C_Rf_ bond yielding intermediates (***I2*** and I2*) with square planar geometries for both metal centers. This process can proceed from [AuCl­(AsPh_3_)] or, more rapidly upon AsPh_3_ addition, from [AuCl­(AsPh_3_)_2_], highlighting the catalytic role of AsPh_3_ in the transmetalation.

Based on oxidation state assignment rules and corroborated by orbital localization studies, the formation of dative Rh–Au bonds reflects a one-electron redox process. In the Rf/Cl transmetalation, ***I2*** corresponds to a Rh(0)–Au­(II) formulation, indicating that Au^|^ is oxidized by Rh^|^(CO). Conversely, in the Rf/Pf exchange, the opposite electron flow gives rise to ***I2^∧^***, best described as Rh­(II)–Au(0). This dual behavior is further supported by NBO and SOPT analyses, which show that the oxidized metal center acts as the electron donor in each case.

Finally, we wish to emphasize the distinctive role of AsPh_3_ as a ligand, whose moderate coordinating ability makes its binding reversible under reaction conditions—unlike stronger ligands such as PPh_3_. This reversibility is key to understanding the observed kinetic effects: coordination to Au^|^ in the Rf/Cl system accelerates transmetalation, while dissociation from Rh in the Rf/Pf system (ref 8) has the opposite effect. Beyond the present study, related investigations in our group have shown that the partial dissociation of substoichiometric AsPh_3_ plays a critical role in (a) preventing Au^I^ disproportionation of [Au­(carbene)­(solv)]^+^ active species under catalytic conditions;^35^ (b) enabling the Au-*co*-catalyzed Stille coupling of sterically demanding aryls;^4c^ and, more recently, (c) suppressing undesired side reactivity in fluorinated aryl–alkynyl Stille couplings.^22^ Although phosphines were historically essential to stabilizing organometallic species, their widespread use may have led to the underappreciation of ligands with more labile coordination. As is the case for hemilabile versus rigid chelates, monodentate arsines represent a promising and underexplored alternative when reversible binding may be mechanistically relevant.

## Experimental Section

### General Considerations

All reactions were performed under N_2_ atmosphere. Solvents were purified according to standard procedures. Complexes *trans*-[RhRf­(CO)­(AsPh_3_)_2_] (**1**),^8^ [AuCl­(AsPh_3_)] (**2**),^36^ *trans*-[RhCl­(CO)­(AsPh_3_)_2_] (**3**),^37^ and [AuRf­(AsPh_3_)] (**4**),^4b^ were prepared following literature procedures. AsPh_3_ and 1,3,5-trichloro-2,4,6-trifluorobenzene (C_6_F_3_Cl_3_) are commercially available and were recrystallized prior to use in the kinetic experiments. Technical measurements were performed with equipment of the LTI services or the IU CINQUIMA (both University of Valladolid) unless otherwise stated.

The NMR spectra were recorded on an Agilent 500 MHz instrument. ^1^H NMR and ^19^F NMR spectra were referenced to TMS and CFCl_3_, respectively. Mass spectra were obtained using a Bruker Maxis Impact time-of-flight mass spectrometer coupled with a matrix-assisted laser desorption/ionization (MALDI–TOF) Bruker Autoflex instrument. The elemental analyses were performed with a Carlo Erba 1108 microanalyser (Vigo University, Spain).

Single-crystal X-ray diffraction data were collected in an Agilent Supernova diffractometer with an Atlas CCD area detector. Data collection was performed with Mo–Kα radiation (λ = 0.71073 Å). Data integration, scaling and empirical absorption correction was performed using the CrysAlisPro program package.^38^ The structure was solved with ShelxT,^39^ and refined with ShelxL,^40^ within the Olex2 software.^41^

No uncommon hazards are noted.

### Characterization of the Fluorinated Complexes

#### trans-[RhRf­(CO)­(AsPh_3_)_2_] (**1**)

NMR data (CD_2_Cl_2_, 293 K): ^19^F δ – 86.25 (d, ^3^ *J*_*Fo‑Rh*_ = 9.5 Hz, 2F_o_), – 122.11 (s, 1F_p_). ^1^H δ 7.54 (m, AsPh_3_, 12H), 7.45–7.30 (m, AsPh_3_, 18H). Anal. Calcd for C_43_H_30_As_2_Cl_2_F_3_ORh: C, 54.75; H, 3.21. Found: C, 54.96; H, 3.03.

#### [AuRf­(AsPh_3_)_2_] (**4**)

NMR data (CD_2_Cl_2_, 293 K): ^19^F δ – 90.20 (s, 2F_o_), – 116.92 (s, 1F_p_). ^1^H δ 7.65–7.50 (m, AsPh_3_, 15H). Anal. Calcd for C_24_H_15_AsAuCl_2_F_3_: C, 41.00; H, 2.15. Found: C, 40.90; H, 2.03.

### Kinetic Studies

Kinetic experiments were monitored by ^19^F NMR. NMR tubes were charged with **1** (4.71 mg, 5.00 × 10^–3^ mmol), **2** (2.69 mg, 5.00 × 10^–3^ mmol) and C_6_F_3_Cl_3_ (0.78 mg, 3.32 × 10^–3^ mmol) as the internal reference. Subsequently, previously cooled CD_2_Cl_2_ (0.50 mL), containing the appropriate amount of dissolved AsPh_3_ depending on the experiment, was added and the tube was then placed into a thermostated probe in the NMR spectrometer. The temperature of the sample (273 K) was confirmed using methanol as a chemical shift thermometer.^42^ Five minutes were allowed for temperature equilibration. Then, concentration–time data were obtained from the integrals of the F_*ortho*_ signals of **1** and **4**.

The experimental data collected from the individual reactions with varying concentrations of AsPh_3_, were simultaneously fitted to the kinetic model shown in Scheme 1 by nonlinear least-squares (NLLS) regression, using the COPASI software.^12^

### Computational Methods

DFT calculations reported in this work were carried out using the dispersion-corrected hybrid functional ωB97X-D,^43^ implemented in the Gaussian09 software.^44^ To describe the C, As, and H atoms, the double-ζ basis set 6–31G­(d,p) was employed, whereas the same basis set with added diffuse functions was used for the more electronegative O, Cl, and F atoms. The Rh and Au metal centers were described using the effective core potential LANL2DZ,^45^ including an *f-*polarization function (exponents: 1.350 for Rh and 1.050 for Au).^46^ Geometry optimizations were performed in vacuum without imposing any constraints, and their nature was further assessed through vibrational frequency analysis. As expected, all the energy minima were confirmed to display only real vibrational frequencies, whereas transition states exhibited one single imaginary frequency. For the latter, geometry relaxations along the reaction coordinate were also carried out to confirm that they connect the corresponding reaction energy minima. Solvent effects were accounted for via single-point calculations at the vacuum-optimized geometries using the SMD solvation model and the solvent employed in experiments, *i.e*. CH_2_Cl_2_ (ε = 8.93).^33^

Selected bonding interactions were investigated by means of NBO and SOPT analyses.^48^ The topology of the electron density was analyzed by means of QTAIM,^28^ as implemented in the Multiwfn package (version 3.7),^50^ using the inherited wave function from the geometry optimizations. The relevant molecular orbitals were localized using the Pipek–Mezey method,^31^ ^31^ as implemented in the MOKIT Python package.^51^

## Supplementary Material



## Data Availability

Additionally, all the DFT-optimized structures reported in this work are openly accessible from the ioChem-BD repository at the following link: https://iochem-bd.bsc.es/browse/handle/100/342841.

## Notes

NMR spectra, kinetic details (experiments and simulations), computational specifics, and crystallographic information (PDF)
