# Supplementary material for: Opposite Effects of Added AsPh3 Reveal a Drastic Mechanistic Switch in RhI/AuI Transmetalations via Rh–Au Bonded Intermediates
Source: Inorg Chem. 2025 Jun 26;64(27):13729–39. doi: 10.1021/acs.inorgchem.5c01081 (PMC12264965; doi:10.1021/acs.inorgchem.5c01081)
Supplement: Supplementary file 1 [file ic5c01081_si_001.pdf]

Supporting Information

Opposite Effects of Added $AsPh_3$ Reveal a Drastic Mechanistic Switch in Rh^{I}/Au^{I} Transmetalations via Rh–Au Bonded Intermediates

Marconi N. Peñas-Defrutos,[a]* *Camino Bartolomé,*[a] *Max García-Melchor,*[b,c,d] *Pablo Espinet*[a]*

[a] IU CINQUIMA/Química Inorgánica, Facultad de Ciencias, Universidad de Valladolid, E–47071 Valladolid, Spain

[b] School of Chemistry, CRANN and AMBER Research Centres, Trinity College Dublin, Dublin 2, Ireland

[c] Center for Cooperative Research on Alternative Energy (CIC EnergiGUNE), Basque Research and Technology Alliance (BRTA), 01510 Vitoria-Gasteiz, Spain;

[d] IKERBASQUE, Basque Foundation for Science, 48009 Bilbao, Spain

E–mail: marconi_44@hotmail.com (M. N. P.-D.) espinet@qi.uva.es (P. E)

Contents

General section and characterization of the complexes S2

NMR spectra S3

Kinetic experiments for Rf/Cl exchange S6

Kinetic simulations with COPASI software S10

Computational section S12

X-ray diffraction details S15

Notes and references S17

Experimental general section and characterization of the complexes

All reactions were performed under N_2 atmosphere. Solvents were purified according to standard procedures.[1] Complexes *trans*-[RhRf(CO)(AsPh$_3$)$_2$] (**1**) (Rf = 3,5–$C_6Cl_2F_3$),[2] [AuCl(AsPh$_3$)] (**2**),[3] *trans*-[RhCl(CO)(AsPh$_3$)$_2$] (**3**),[4] and [AuRf(AsPh$_3$)] (**4**),[5] were prepared according to the literature procedures. Triphenylarsine (AsPh$_3$) and 1,3,5-trichloro-2,4,6-trifluorobenzene ($C_6F_3Cl_3$) are commercially available and were recrystallized for its use in the kinetic experiments. The technical measurements were carried out with equipment of the LTI services or the IU CINQUIMA (both of the University of Valladolid) unless otherwise stated.

The NMR spectra were recorded with Agilent 500 NMR instrument. ^{1}H NMR and ^{19}F NMR spectra are referred to TMS and $CFCl_3$, respectively. The mass spectra were recorded with a Bruker Maxis Impact flight time mass spectrometer coupled to matrix-assisted laser desorption ionization (MALDI–TOF) Bruker Autoflex. The elemental analyses were performed with a Carlo Erba 1108 microanalyser (by Vigo University, Spain).

Data of *trans*-[RhRf(CO)(AsPh$_3$)$_2$] (1):

^{19}F NMR (470.15 MHz, CD_2Cl_2, 293 K): δ –86.25 (d, $^3J_{Fo\text{-}Rh}$ = 9.5 Hz, 2F$_o$), –122.11 (s, 1F$_p$).

^{1}H NMR (499.72 MHz, CD_2Cl_2, 293 K): δ 7.54 (m, AsPh$_3$, 12H), 7.45-7.30 (m, AsPh$_3$, 18H).

Analysis calculated for $C_{43}H_{30}As_2Cl_2F_3ORh$: C, 54.75; H, 3.21. Found: C, 54.96; H, 3.03.

Data of [AuCl(AsPh$_3$)] (2):

^{1}H NMR (499.72 MHz, CD_2Cl_2, 293 K): δ 7.65-7.45 (m, AsPh$_3$, 15H).

Analysis calculated for $C_{18}H_{15}AsAuCl$: C, 40.14; H, 2.81. Found: C, 40.25; H, 2.60.

Data of *trans*-[RhCl(CO)(AsPh$_3$)$_2$] (3):

^{1}H NMR (499.72 MHz, CD_2Cl_2, 293 K): δ 7.70 (m, AsPh$_3$, 12H), 7.50-7.40 (m, AsPh$_3$, 18H).

Analysis calculated for $C_{37}H_{30}As_2ClORh$: C, 57.06; H, 3.88. Found: C, 57.30; H, 3.76.

Data of [AuRf(AsPh$_3$)] (4):

Suitable single crystals for X-ray crystallography were obtained by layering hexane in a dichloromethane solution of **4** (Figure S5)

^{19}F NMR (470.15 MHz, CD_2Cl_2, 293 K): δ –90.20 (s, 2F$_o$), –116.92 (s, 1F$_p$).

^{1}H NMR (499.72 MHz, CD_2Cl_2, 293 K): δ 7.65-7.50 (m, AsPh$_3$, 15H).

Analysis calculated for $C_{24}H_{15}AsAuCl_2F_3$: C, 41.00; H, 2.15. Found: C, 40.90; H, 2.03.

Data of [AuCl(AsPh$_3$)$_2$] (5):

In a solution of **2** + 10 mol% of AsPh$_3$ in CH_2Cl_2 we managed to detect **5** by mass spectrometry.

HRMS (MALDI-TOF, *m/z*): calcd. for $C_{36}H_{30}As_2Au$ $[M – Cl]^+$: 809.0440; found: 809.0422.

NMR spectra

^{19}F NMR spectrum of complex *trans*-$[RhRf(CO)(AsPh_3)_2]$ (**1**) in CD_2Cl_2 at 293 K.

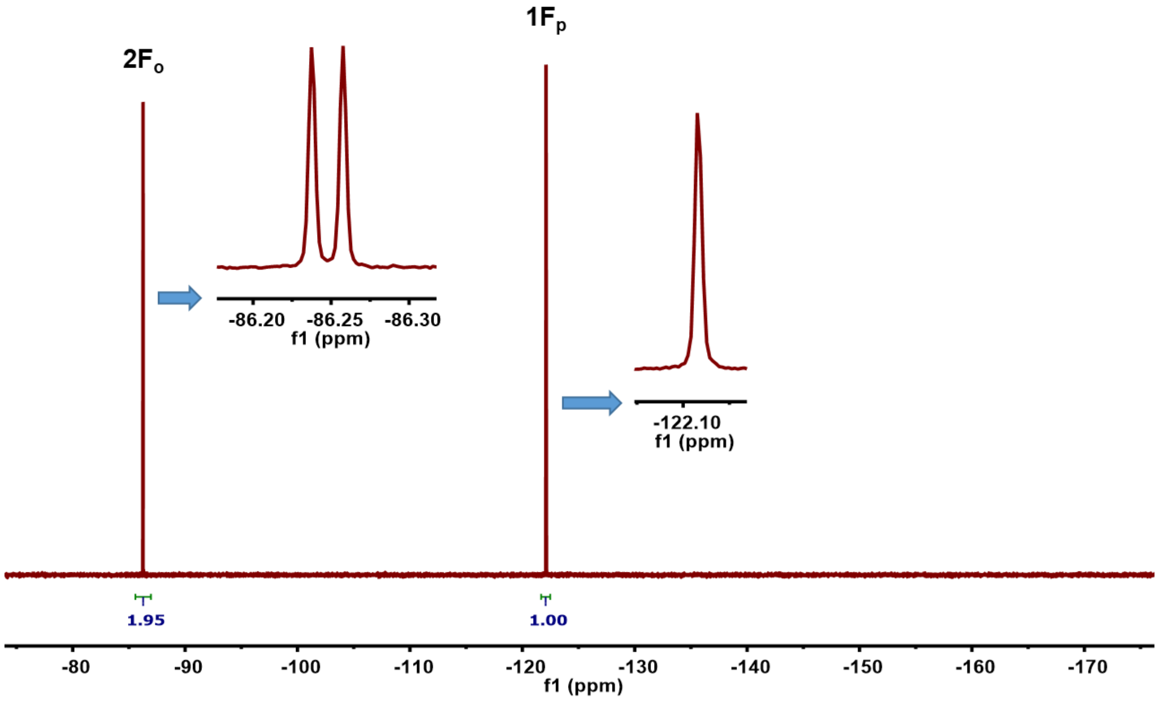

^{1}H NMR spectrum of complex *trans*-$[RhRf(CO)(AsPh_3)_2]$ (**1**) in CD_2Cl_2 at 293 K. Expansion of $AsPh_3$ signals.

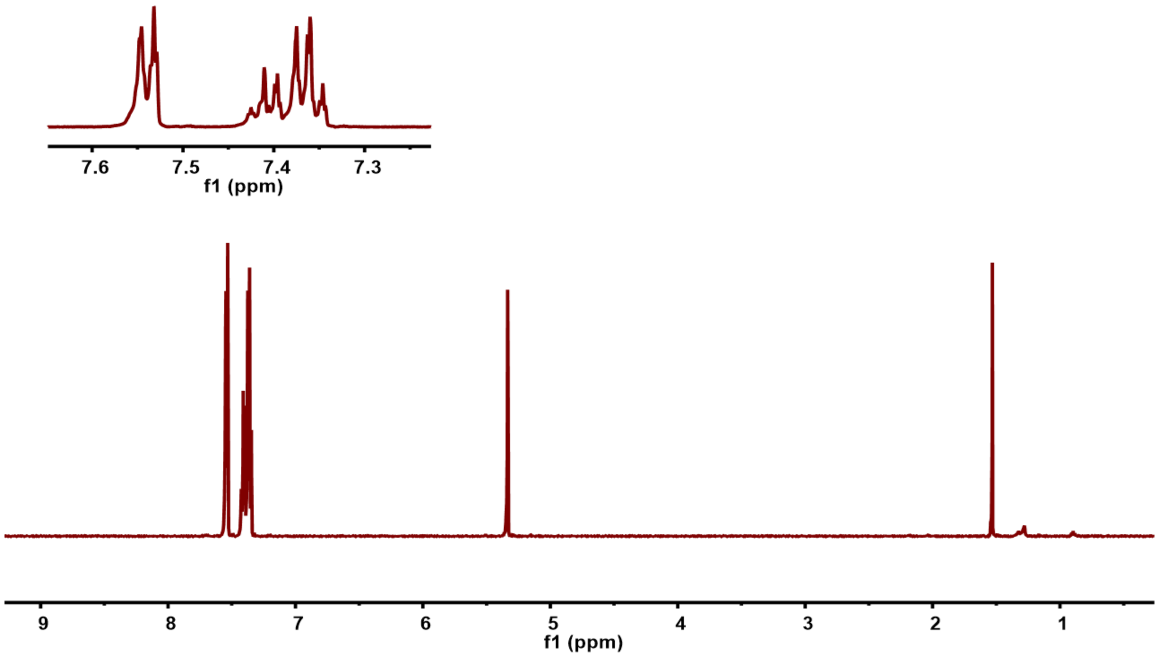

^{19}F NMR spectrum of complex [AuRf(AsPh$_3$)] (**4**) in CD_2Cl_2 at 293 K.

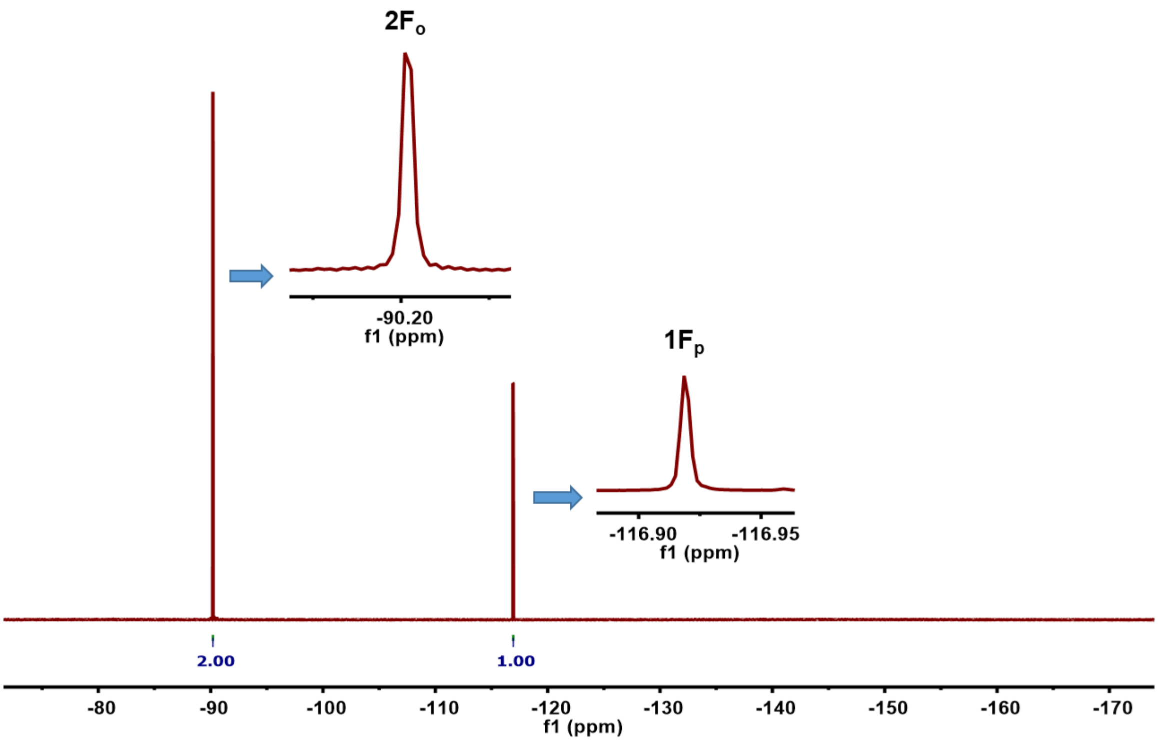

^{1}H NMR spectrum of complex [AuRf(AsPh$_3$)] (**4**) in CD_2Cl_2, 293 K. Expansion of AsPh$_3$ signals.

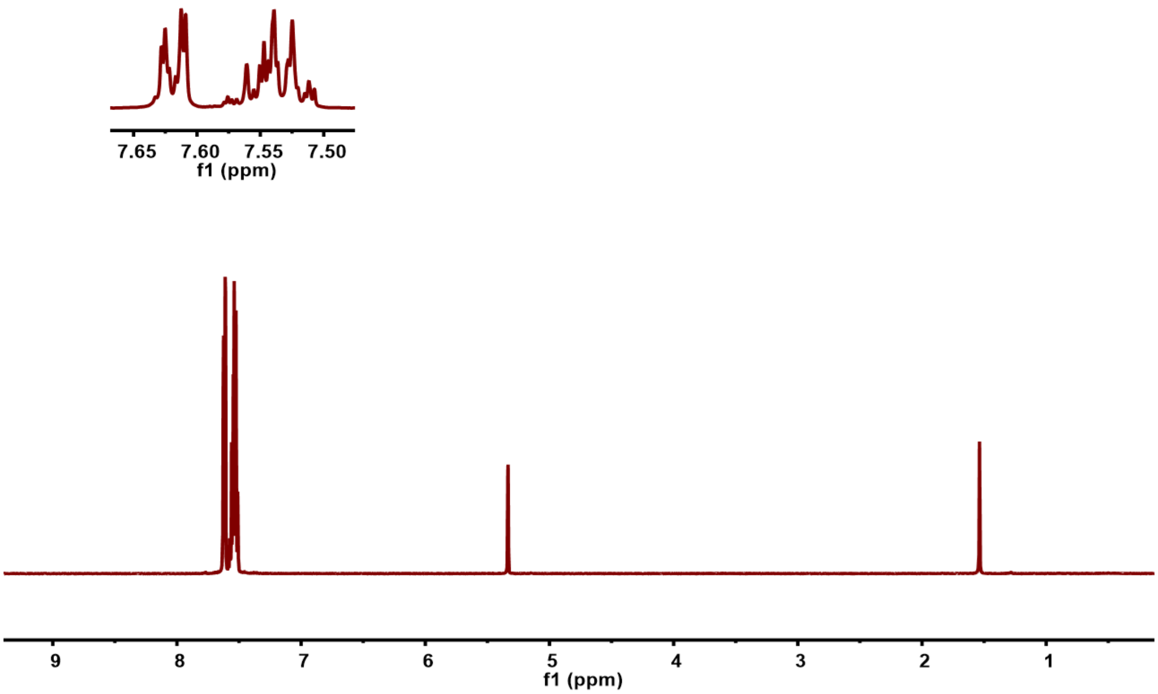

^{1}H NMR spectrum of complex [AuCl($AsPh_3$)] (**2**) in CD_2Cl_2 at 293 K. Expansion of $AsPh_3$ signals.

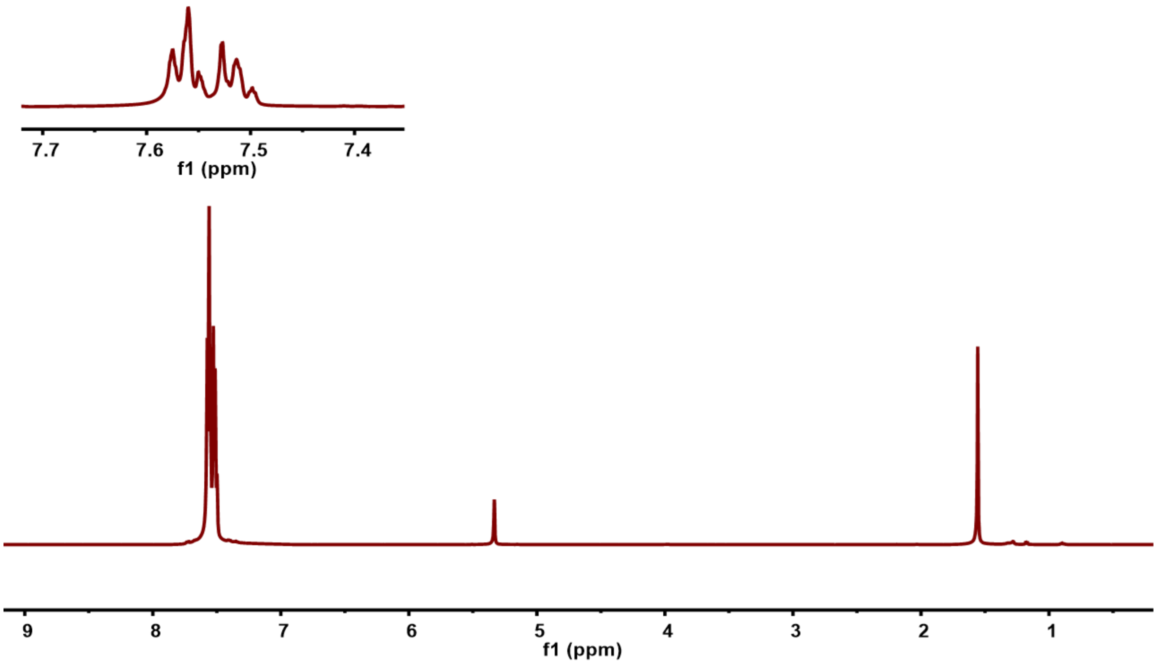

^{1}H NMR spectrum of complex *trans*-[RhCl(CO)($AsPh_3$)$_2$] (**3**) in CD_2Cl_2 at 293 K. Expansion of $AsPh_3$ signals.

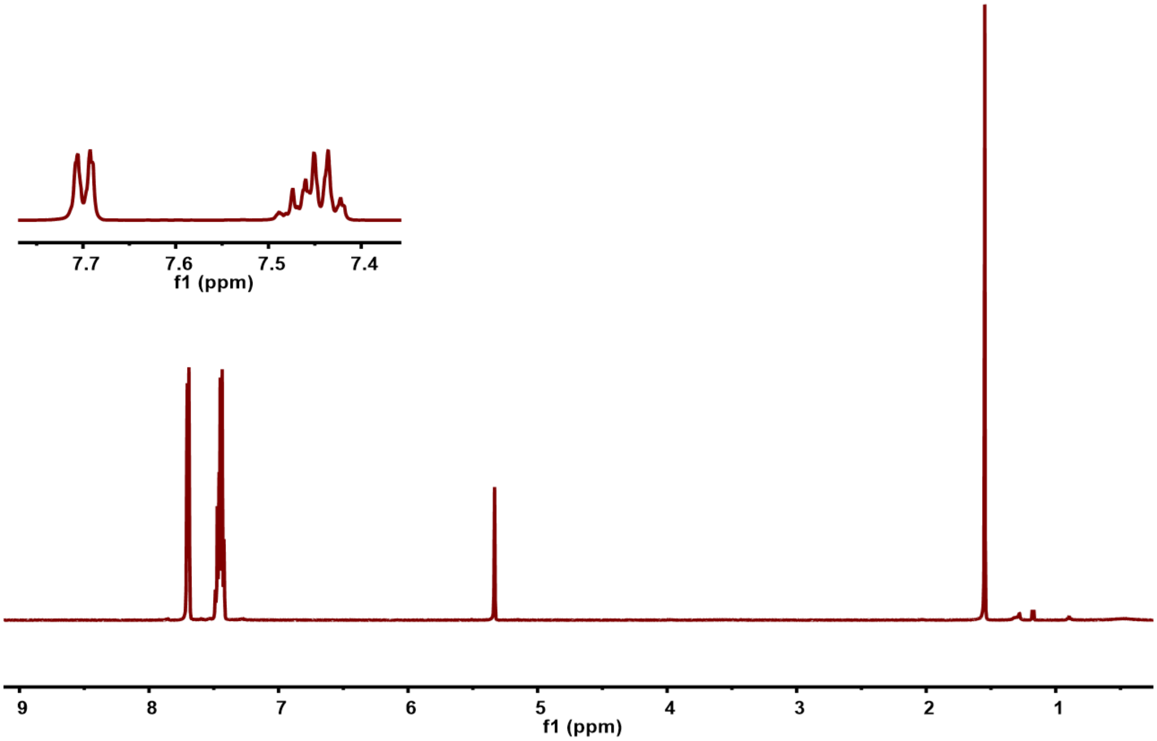

Kinetic experiments for Rf/Cl exchange (Scheme S1)

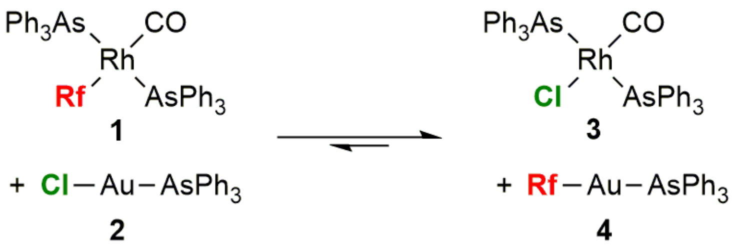

Scheme S1. Rf/Cl exchange reaction (Rf = $C_6F_3Cl_2$-3,5)

Kinetic experiments were monitored by ^{19}F NMR. NMR tubes were charged with *trans*-[RhRf(CO)($AsPh_3$)$_2$] (**1**) (4.71 mg, 5.00 × 10^{-3} mmol), the gold complex [AuCl($AsPh_3$)] (**2**) (2.69 mg, 5.00 × 10^{-3} mmol) and 1,3,5-trichloro-2,4,6-trifluorobenzene[6] (0.78 mg, 3.32 × 10^{-3} mmol) as internal reference. Subsequently, previously cooled CD_2Cl_2 (0.50 mL) with the corresponding amount of dissolved $AsPh_3$, depending on the experiment, was added and the tube was placed into a thermostated probe in a Agilent 500 NMR instrument apparatus. The temperature of the sample (273 K) was confirmed using methanol as chemical shift thermometer.[7]

Five minutes were left for temperature equilibration. Then, concentration-time data were obtained from the integrals of the F_{ortho} signals of *trans*-[RhRf(CO)($AsPh_3$)$_2$] (**1**) and [AuRf($AsPh_3$)] (**4**). ^{19}F NMR spectra were recorded each 30 seconds, using 1 scan (90° angle) acquisitions. The influence of the reagents and the effect of addition of free ligand were studied.

In the case of experiments with free ligand, 0.50 mL of previously prepared CD_2Cl_2 solutions of $AsPh_3$ with different concentrations (1.0 × 10^{-3}, 6.0 × 10^{-3}, 5.0 × 10^{-2} and 2.0 × 10^{-1} mol × L^{-1}) were added to the reagents.

The equilibrium constant was stablished studying also the inverse reaction until steady concentrations were observed (Figure S1). A NMR tube was charged with *trans*-[RhCl(CO)($AsPh_3$)$_2$] (**3**) (3.75 mg, 5.00 × 10^{-3} mmol), the gold complex [AuRf($AsPh_3$)] (**4**) (3.51 mg, 5.00 × 10^{-3} mmol) and 1,3,5-trichloro-2,4,6-trifluorobenzene (0.78 mg, 3.32 × 10^{-3} mmol) as internal reference. Then CD_2Cl_2 (0.50 mL) was added.

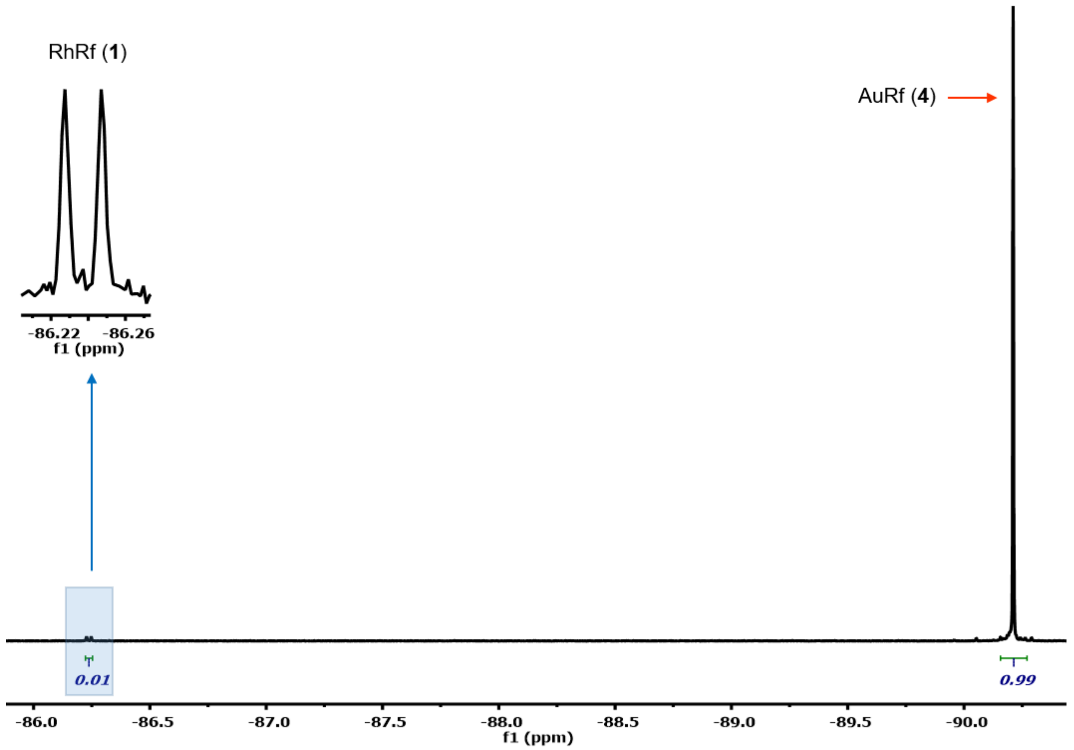

Figure S1. ^{19}F NMR spectrum of the stoichiometric reaction between **3** and **4** in CD_2Cl_2 after 1 day at room temperature (equilibrium already reached). F_{ortho} region with integrated signals.

Kinetic experiments with different ratio of the reagents

Two experiments were prepared in order to prove that the reaction follows first order kinetics in both metal reagents.

A) Reaction of *trans*-[RhRf(CO)$(AsPh_3)_2$] (**1**) (0.010 mol × L^{-1}) and [AuCl$(AsPh_3)$] (**2**) (0.020 mol × L^{-1}). Excess of gold complex.

B) Reaction of *trans*-[RhRf(CO)$(AsPh_3)_2$] (**1**) (0.020 mol × L^{-1}) and [AuCl$(AsPh_3)$] (**1**) (0.010 mol × L^{-1}). Excess of rhodium complex.

Table S1 collects the initial rates and Figure S2 summarizes the kinetic data of experiments A and B compared with the 1:1 experiment (orange series in Figure 2). Note that both 2:1 and 1:2 ratios double the rate observed in the 1:1 reaction.

Table S1. Initial rate (r_0) for the experiments summarized in Figure S2 and linear fitting parameters up to 10% conversion (T = 273 K).

**Au Reagent** **[Au]$_0$** (mol × L^{-1})	**Rh Reagent** **[Rh]$_0$** (mol × L^{-1})	**Slope (*r_0*)** (mol × L^{-1} × s^{-1})	**Intercept** (mol × L^{-1})	**R^2**
**[1]** = 0.010	**[2]** = 0.010	2.16×10^{-7}	9.71×10^{-5}	0.999
**[1]** = 0.020	**[2]** = 0.010	4.38×10^{-7}	4.57×10^{-5}	0.997
**[1]** = 0.010	**[2]** = 0.020	3.95×10^{-7}	7.34×10^{-5}	0.996

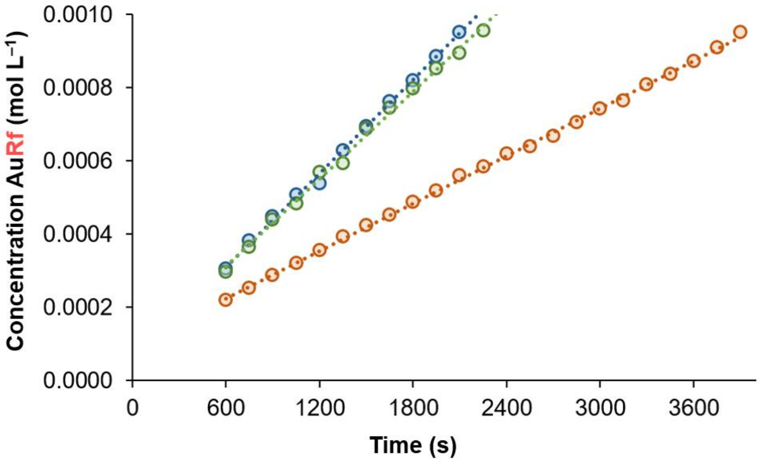

Figure S2. Linear fitting of concentration data in experiments **A** (blue) and **B** (orange) compared with the stoichiometric reaction (orange).

Kinetic experiments with different amounts of free $AsPh_3$ added

Figure 2 collects the concentration *vs*. time experimental data obtained by monitoring the formation of the product [AuRf($AsPh_3$)] (**4**) by means of ^{19}F NMR in CD_2Cl_2 at 273 K, with different amounts of free ligand added. Initial concentrations of the reactants: $[Rh]_0 = [Au]_0 = 1.0 \times 10^{-2}$ mol L^{-1}.

Table S1 collects the initial rates and Figure S3 summarizes the kinetic data, of experiments with 0 mol%, 10 mol%, 60 mol% and 500 mol% of added $AsPh_3$.

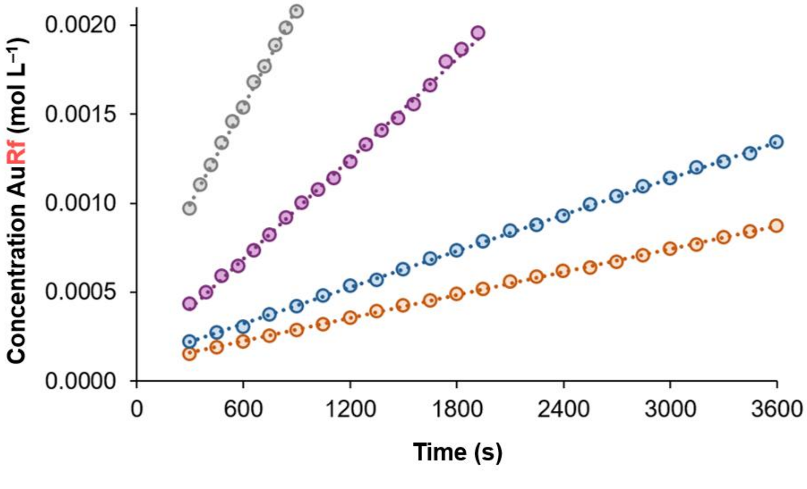

Figure S3. Linear fitting of concentration *vs.* time data in Rh:Au 1:1 experiments with 0 mol% (orange), 10 mol% (blue), 60 mol% (violet) and 500 mol% (green) of free $AsPh_3$ added.

Table S2. Initial rate (r_0) for the experiments summarized in Figure S2 and linear fitting parameters (T = 273 K). Data up to 10% conversion for orange and blue traces, up to 15% for violet trace and 20% for grey trace.

**Colour**	**$[AsPh_3]_0$** (mol × L^{-1})	**Slope (r_0)** (mol × L^{-1} × s^{-1})	**Intercept** (mol × L^{-1})	**R^2**
Orange	0	2.16×10^{-7}	9.71×10^{-5}	0.999
Blue	0.001	3.49×10^{-7}	1.06×10^{-4}	0.999
Violet	0.006	9.23×10^{-7}	1.32×10^{-4}	0.996
Grey	0.050	1.84×10^{-6}	4.46×10^{-4}	0.998

Kinetic experiments in acetone

Figure S4 collects the concentration *vs.* time experimental data obtained by monitoring the formation of the product $[AuRf(AsPh_3)]$ (**4**) by means of ^{19}F NMR in acetone-d_6 at 273 K, without free ligand added (orange trace) and with 10 mol% of $AsPh_3$ (blue trace, $[L]_0 = 1.0 \times 10^{-3}$ mol L^{-1}). Initial concentrations of the reactants: $[Rh]_0 = [Au]_0 = 1.0 \times 10^{-2}$ mol L^{-1}.

Least-squares fitting affords r_0 values of 3.80×10^{-6} and 1.19×10^{-5} mol × L^{-1} × s^{-1} respectively, meaning that the catalyzed reaction is 3.1 times faster than the reaction without added $AsPh_3$.

* Note that the uncatalyzed reaction is faster than the analogous in dicholoromethane (Figure S2) but solubility issues preclude monitoring at lower temperatures.

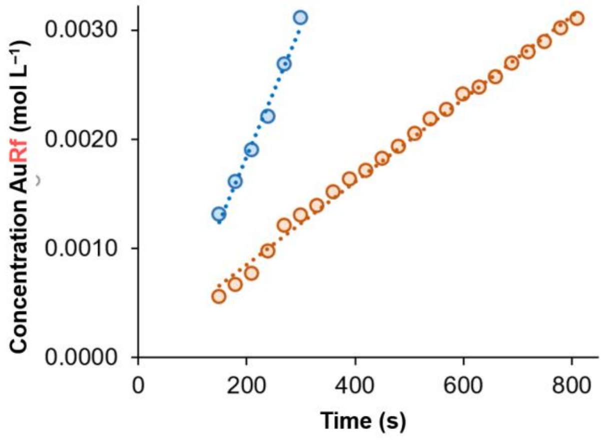

Figure S4. Linear fitting of concentration *vs.* time data in Rh:Au 1:1 experiments with 0 mol% (orange), 10 mol% (blue) of free $AsPh_3$ added in acetone-d_6 at 273K.

Kinetic simulations with COPASI software

The kinetic model shown in Scheme S2 was fitted to the measured concentration *vs.* time experimental data by nonlinear least-squares (NLLS) regression, using the software COPASI.[8] Table S3 summarises the adjusted kinetic constants for the data depicted in Figure 2 (see a particular example in Figure S4). The reversible ligand association pre-equilibrium is a fast process in which the equilibrium constant ($K_{eq\text{-}AsPh3} = k_1 / k_{-1}$) is crucial.

k_A can also be obtained from the experiment without added free $AsPh_3$ by the initial-rate approximation method and agrees with the COPASI fitted value. The experimental equilibrium constant for the transmetalation reaction ($K_{eq} \approx 7 \times 10^3$) allows to calculate k_{-A} considering that $K_{eq} = k_A / k_{-A}$. The value for k_B was calculated from the simultaneous fitting of the experimental data from the 4 individual reactions.

The units of the rate constants are (s^{-1}), (mol^{-1} L s^{-1}) or (mol^{-2} L^2 s^{-1}) for first, second and third order kinetic reactions respectively.

Note that the kinetic model simplifies both pathways to one-step reactions. This simplification is perfectly correct attending to the mechanisms shown in Figures 3 and 9, in which the rate-limiting step in both cases is the oxidative insertion occurring through the first Transition State (***TS1*** and ***TS1**** respectively).

The equation used to calculate the $\Delta G^{\ddagger}$ from the kinetic constants is:[9]

$$k_{kinetic} = \frac{k_B T}{h} e^{-\frac{\Delta G^{\ddagger}}{RT}}$$

$\boldsymbol{k_B}$ = Boltzmann constant

$\boldsymbol{h}$ = Plank constant

$\boldsymbol{T}$ = temperature, in our case 273 K

The equation used to calculate the ΔG_0 from the equilibrium constants is:

$$\Delta G_0 = -RT ln K_{eq}$$

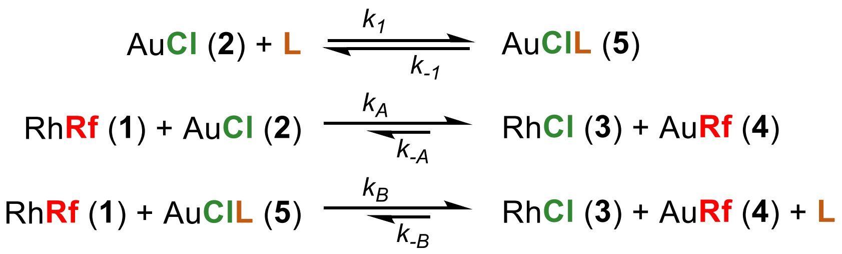

Scheme S2. Kinetic model for the COPASI fitting (see Scheme 1).

Table S3. Fitted kinetic constants. Starting conditions: [**1**]$_0$ = [**2**]$_0$ = 0.010 mol L^{-1}; [**3**]$_0$ = [**4**]$_0$ = [**5**]$_0$ = 0 mol L^{-1}; and [**L**]$_0$ depending on the case. Adjusted $K_{eq\text{-}AsPh3} = k_1 / k_{-1} = 1.21 \times 10^2$ mol^{-1} L.

k_1	k_{-1}	k_A	k_{-A}	k_B	k_{-B}
4.809×10^2	2.257×10^0	2.602×10^{-3}	3.700×10^{-7}	3.110×10^{-2}	1.100×10^{-3}

* Errors for adjusted k_A and k_B are 1.36% and 1.38% respectively. Best value of fitting = 4.3×10^{-7}

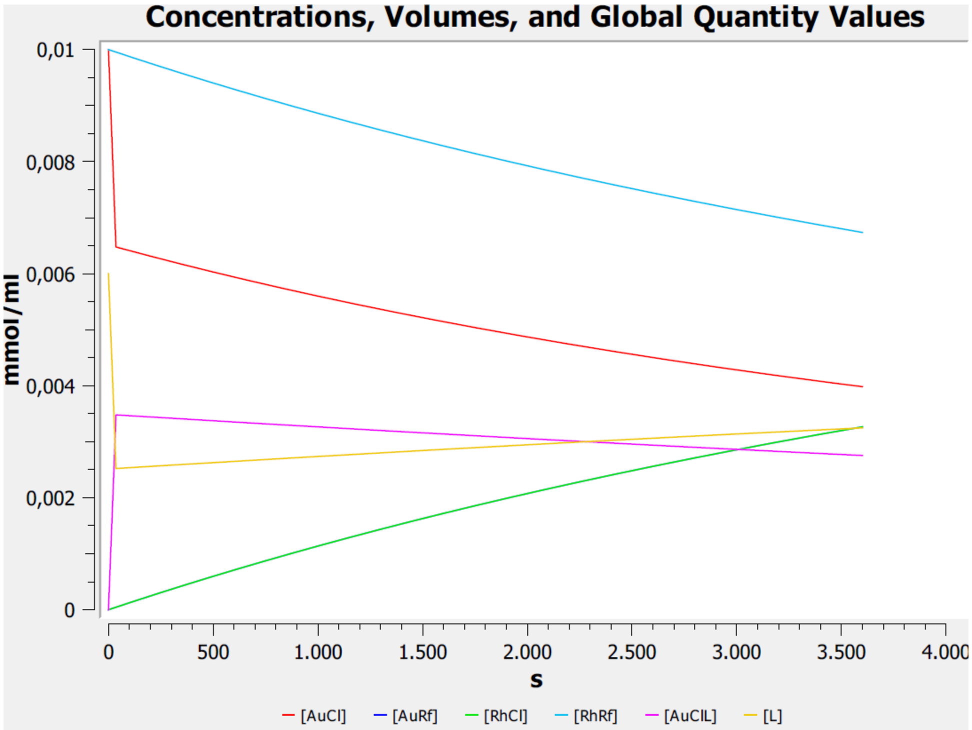

Figure S5. Concentration-time data plot of all species simulated for the experiment with 60 mol% of free added $AsPh_3$ (pink trace in Figure 2 corresponds to the green trace in this figure). Note that only complexes **1** and **4** (blue and green respectively here) can be monitored by ^{19}F NMR.

Computational section

Density functional theory (DFT) calculations reported in this work were carried out using the dispersion-corrected hybrid exchange-correlation functional ωB97X-D,[10] implemented in the Gaussian09 software.[11] The choice of this level of theory is based on the satisfactory results obtained in our previous theoretical study on related Rh^{I}/Au^{I} transmetalations.[2] To describe the C, As and H atoms, the double-ζ basis set 6-31G(d,p) was employed, whereas the same basis set with added diffuse functions was used to describe the more electronegative O, Cl and F atoms. For Rh and Au metals, the effective core potential LANL2DZ[12] its associated double-ζ basis set plus *f*-polarization functions (exponents: 1.350 for Rh and 1.050 for Au)[13] were used. With this methodology, the geometry of the stationary points was optimized in vacuum without imposing any constraints, and their nature was further assessed through vibrational frequency analysis. As expected, all the energy minima were confirmed to display only real vibrational frequencies, whereas transition states were found to exhibit one single imaginary frequency. The latter were also confirmed to connect the expected energy minima by relaxing the transition state geometry along the reaction coordinate, following the eigenvector associated to the imaginary frequency.

Solvent effects were accounted for via single-point calculations at the optimized geometries in vacuum using the SMD solvation model and the same solvent employed in experiments, *i.e.* CH_2Cl_2 ($\varepsilon = 8.93$).[14]

All the DFT data underlying this work, including the Cartesian coordinates of the modelled structures and energies, are available at the following ioChem-BD online data set:

https://iochem-bd.bsc.es/browse/review-collection/100/342841/2e7cf9a3d59570eca6e1bab3

Selected bonding interactions were studied by means of natural bond orbital (NBO) and second order perturbation theory (SOPT) analyses.[15] We also investigated the main donations in ***TS1***^ and ***I2***^, reported in our previous article.[2] Figures 7, 8 and S7 depict the most important donor-acceptor interactions in the different transitions states and Rh–Au bonded intermediates. Table S4 summarizes the orbitals involved in each case.

The topology of the electron density was analysed by means of Quantum Theory of Atoms in Molecules (QTAIM)[16] for intermediates ***I2*** and ***I2***^, as implemented in the Multiwfn package (version 3.7),[17] using the inherited wavefunction from the geometry optimizations using Gaussian09. In both cases, a bond critical point (BCP) was identified between Rh and Au metal atoms (Figure 6).

The non-bonding valence d orbitals of rhodium in ***I2*** were localized using the Pipek–Mezey method,[18] implemented via the MOKIT Python package,[19] following visual inspection of the molecular orbitals.

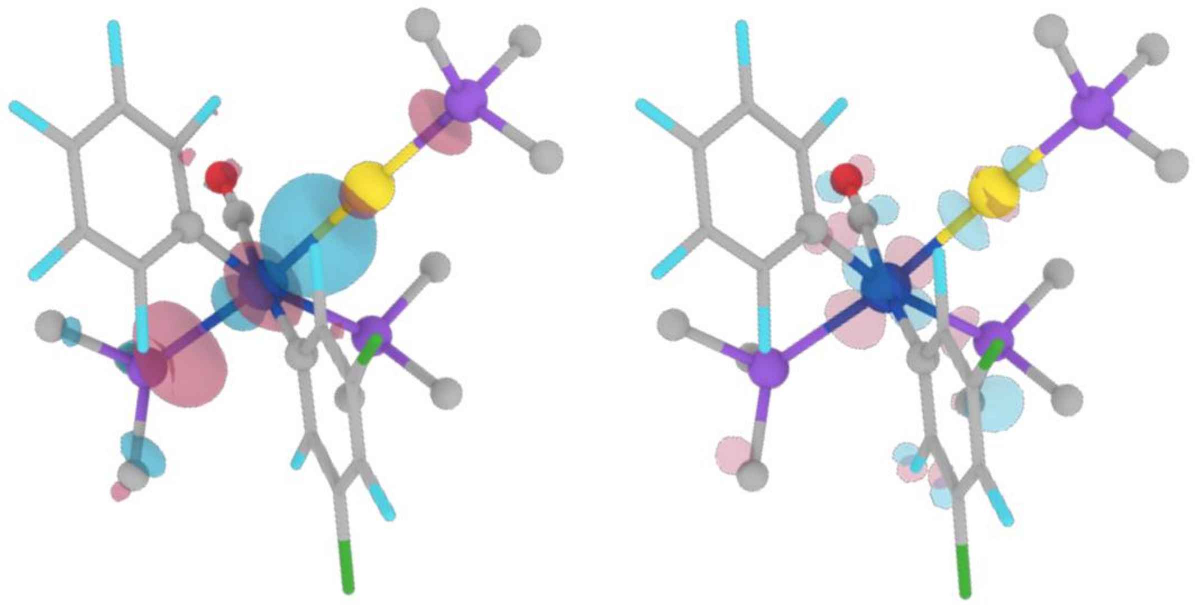

Figure S6. Isosurfaces (isovalue = 0.05 a.u.) of frontier molecular orbitals (left: HOMO; right: LUMO) from ***I2^***. Ph groups in the $AsPh_3$ ligands have been omitted for clarity. F: light blue; Cl: green; As: violet; O: red.

Table S4. Selected donor-acceptor interactions by means of NBO analysis for ***TS1***, ***TS1***^, ***TS1****, ***I2***, ***I2***^, and ***I2****. *LP*, *LV*, *BD* and *BD** stand for Lone Pair, Low Valence, Bonding and Antibonding orbitals respectively. SOPT energies in kcal mol^{-1}.

	Donor	*Contribution*	*Acceptor*	*Contribution*	*Energy*
TS1<br>Rf–Au → Rh	BD Au–C_{Rf}	88% C s (28%) p (72%)<br>12% Au s (95%) p (1%) d (4%)	BD* Rh–C_{CO}	73% Rh s (46%) d (54%)<br>27% C s (65%) p (35%)	100.4[a]
TS1^<br>Pf–Rh → Au	BD Rh–C_{Pf}	83% C s (28%) p (72%)<br>17% Rh s (56%) d (44%)	LV Au	Au s (93%) d (7%)	129.3[a]
TS1*<br>Rf–Au → Rh	BD Au–C_{Rf}	84% C s (29%) p (71%)<br>16% Au s (95%) d (5%)	BD* Rh–C_{CO}	64% Rh s (10%) d (90%)<br>36% C s (65%) p (35%)	68.6[b]
TS1*<br>Rf–Au → Rh	BD Au–C_{Rf}	84% C s (29%) p (71%)<br>16% Au s (95%) d (5%)	LV Rh	Rh s (89%) d (11%)	35.3
I2<br>**Au → Rh**	LP Au	Au s (7%) d (93%)	BD* Rh–C_{CO}	73% Rh s (46%) d (54%)<br>27% C s (65%) p (35%)	33.8[c]
I2^<br>**Rh → Au**	LP Rh	Rh s (1%) d (99%)	BD* Au–As	77% Au s (92%) d (8%)<br>23% As s (33%) p (67%)	45.0[c]
I2*<br>**Au → Rh**	LP Au	Au s (6%) d (94%)	BD* Rh–C_{CO}	72% Rh s (44%) d (56%)<br>28% C s (64%) p (36%)	23.7[b]

[a] See Figure 6. [b] See Figure S6. [c] See Figure 7.

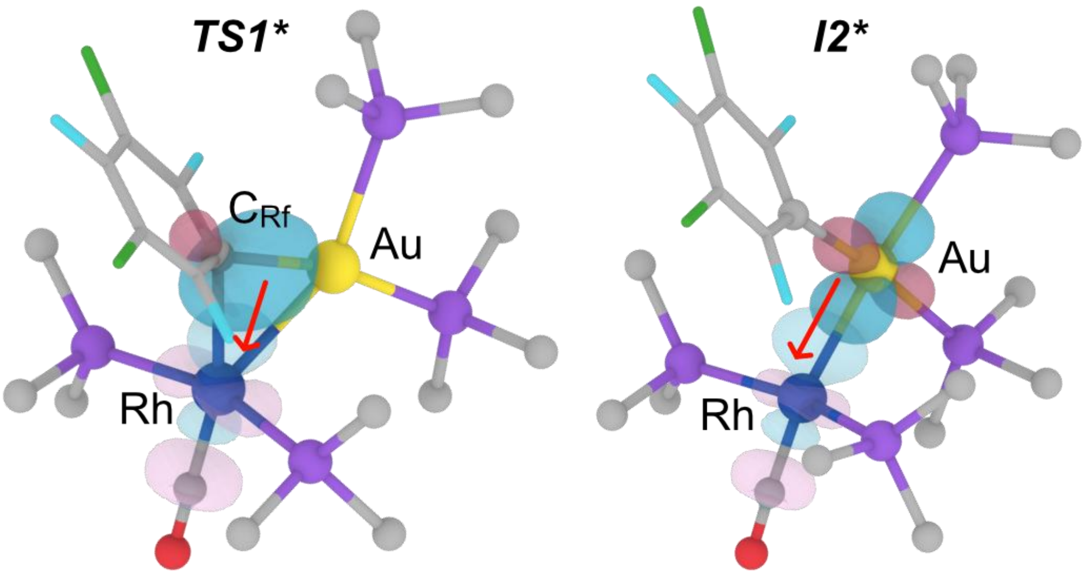

Figure S7. Isosurfaces (isovalue = 0.07 a.u.) of selected NBOs in ***TS1**** (left) and ***I2**** (right). The red arrow denotes the sense of electron donation. Ph groups in the $AsPh_3$ ligands and the dissociated chloride have been omitted for clarity. F: light blue; Cl: green; As: violet; O: red.

The activation strain analyses (ASA),[20] were performed on the ***TS1*** and ***TS1***^ structures via single-point calculations on the individual fragments consisting in the reactants used in each case. Distortion and interactions energies gathered in Table S5 are derived from the following equations:

$$E_{int} = E_{TS}^{opt} - E_{[Au]}^{strain} - E_{[Rh]}^{strain}$$

$$E_{dist[Au]} = E_{[Au]}^{strain} - E_{[Au]}^{opt} \qquad E_{dist[Rh]} = E_{[Rh]}^{strain} - E_{[Rh]}^{opt}$$

$$E_{dist} = E_{dist[Au]} + E_{dist[Rh]}$$

E refers to the energy in the gas phase, the subscripts denote the assigned fragments (specifically *trans*-$[RhRf(CO)(AsPh_3)_2]$ in both cases, and either $[AuCl(AsPh_3)]$ or $[AuPf(AsPh_3)]$ for ***TS1*** and ***TS1***^ respectively) while the superscripts indicate *strain* if the geometry was taken from the optimized transition state or alternatively, *opt* if it is the subsequently relaxed geometry.

Table S5. Summary of the interaction and distortion energy contributions obtained by means of ASA for ***TS1*** and ***TS1***^ supporting the discussion raised in the main text. Energies in kcal mol^{-1}.

	TS1	***TS1***^
E_{int}	64.5	71.3
$E_{dist\,[Au]}$	28.8	32.8
$E_{dist\,[Rh]}$	23.0	23.8
E_{dist}	51.8	56.6

X-ray diffraction details

Refinement of the X-ray structure gives the residuals shown in Table S6.

The crystal was attached to a glass fiber and transferred to an Agilent Supernova diffractometer with an Atlas CCD area detector. Data collection was performed with Mo-Kα radiation (λ = 0.71073 Å). Data integration, scaling and empirical absorption correction was carried out using the CrysAlisPro program package.[21] The crystal was kept at 294 K during data collection. Using Olex2,[22] the structure was solved with the ShelxT,[23] and refined with ShelxL.[24] The non-hydrogen atoms were refined anisotropically and hydrogen atoms were placed at idealized positions and refined using the riding model.

CCDC 2045995 and 2108472 contain the supporting crystallographic data for this article. These data can be obtained free of charge at www.ccdc.cam.ac.uk/conts/retrieving.html [or from the Cambridge Crystallographic Data Centre, 12, Union Road, Cambridge CB2 1EZ, UK; fax: (internat.) +44-1223/336-033; E-mail: deposit@ccdc.cam.ac.uk]. Figure S8 shows the molecular structures for complexes **3** and **4**.

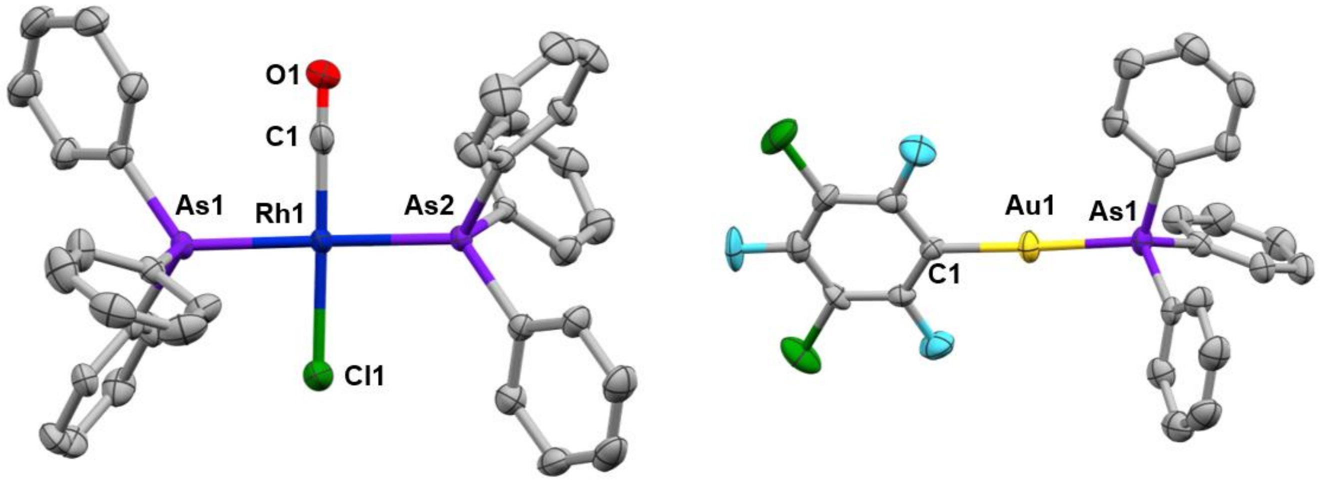

Figure S8. X-ray structures of *trans*-[RhCl(CO)($AsPh_3$)$_2$] (**3**, left) and [AuRf($AsPh_3$)] (**4**, right).

Table S6. Crystal data and structure refinements for **3** and **4**.

	**3**	**4**
Empirical formula	$C_{37}H_{30}As_2ClORh$	$C_{24}H_{15}F_3Cl_2AsAu$
Formula weight	778.81	703.15
Temperature/K	294	294
Crystal system	triclinic	triclinic
Space group	P-1	P-1
a/Å	9.9045(5)	9.1654(4)
b/Å	10.1559(6)	10.8350(5)
c/Å	18.1116(9)	12.0110(7)
α/°	75.446(5)	80.498(4)
β/°	75.692(4)	82.916(4)
γ/°	72.390(5)	85.609(4)
Volume/Å^3	1651.57(17)	1165.51(10)
Z	2	2
ρ_{calc}g/cm^3	1.566	2.004
μ/mm^{-1}	2.614	7.982
F(000)	776.0	664.0
Crystal size/mm^3	0.352 × 0.17 × 0.12	0.246 × 0.128 × 0.043
Radiation	MoKα (λ = 0.71073)	MoKα (λ = 0.71073)
2θ range for data collection/°	6.88 to 58.974	6.924 to 59.126
Index ranges	$-12 \le h \le 12$, $-12 \le k \le 13$, $-17 \le l \le 24$	$-12 \le h \le 12$, $-12 \le k \le 14$, $-14 \le l \le 16$
Reflections collected	11506	8195
Independent reflections	7547 [R_{int} = 0.0261, R_{sigma} = 0.0558]	5320 [R_{int} = 0.0276, R_{sigma} = 0.0627]
Data/restraints/parameters	7547/2/396	5320/0/280
Goodness-of-fit on F^2	1.050	1.047
Final R indexes [I>=2σ (I)]	R_1 = 0.0396, wR_2 = 0.0650	R_1 = 0.0429, wR_2 = 0.0672
Final R indexes [all data]	R_1 = 0.0686, wR_2 = 0.0793	R_1 = 0.0766, wR_2 = 0.0807
Largest diff. peak/hole / eÅ^{-3}	0.50/-0.59	0.63/-0.67

Notes and references

(1) Perrin, D.; Armarego, DW. L. F. *Purification of Laboratory Chemicals*, 3rd ed., Pergamon Press, Oxford, UK, **1988**.

(2) Peñas-Defrutos, M. N.; Bartolomé, C.; García-Melchor, M.; Espinet, P. $Rh^{I}Ar/Au^{I}Ar'$ Transmetalation: A Case of Group Exchange Pivoting on the Formation of M−M′ Bonds through Oxidative Insertion. *Angew. Chem. Int. Ed.* **2019**, *58*, 3501–3505.

(3) Nieto-Oberhuber, C.; López, S.; Echavarren, A. M. Intramolecular [4 + 2] Cycloadditions of 1,3-Enynes or Arylalkynes with Alkenes with Highly Reactive Cationic Phosphine Au(I) Complexes. *J. Am. Chem. Soc.* **2005**, *127*, 6178–6179.

(4) Osborn, J. A.; Wilkinson, G.; Evans, D. *Trans*-Chlorocarbonylbis(Tri-Phenylphosphine)Rhodium and Related Complexes. *Inorg. Synth.* **1968**, *11*, 99–101.

(5) (a) Byabartta, P. Organometallic gold(I)-pentafluorophenyl-P, O, As, S, TPA, dppm, dppe, dppa-coordinating-phosphines: synthesis and detailed spectroscopic characterisation. *Transit. Met. Chem.* **2007**, *32*, 716–726. (b) Pérez-Temprano, M. H.; Casares, J. A.; R. de Lera, A.; Álvarez, R.; Espinet. P. Strong Metallophilic Interactions in the Palladium Arylation by Gold Aryls. *Angew. Chem. Int. Ed*. **2012**, *51*, 4917–4920.

(6) Commercially available. ^{1}F NMR (470.15 MHz, CD_2Cl_2, 273 K): δ –113.20 (s, 3F).

(7) Ammann, C.; Meier, P.; Merbach, A. E. A Simple Multinuclear NMR Thermometer. *J. Magn. Reson.* **1982**, *46*, 319–321.

(8) Complex pathway simulator: Hoops, S.; Sahle, S.; Gauges, R.; Lee, C.; Pahle, J.; Simus, N.; Singhal, M.; Xu, L.; Mendes, P.; Kummer, U. COPASI—a COmplex PAthway Simulator. *Bioinformatics*, **2006**, *22*, 3067–3074.

(9) Kozuch, S.; Shaik, S. How to Conceptualize Catalytic Cycles? The Energetic Span Model. *Acc. Chem. Res*. **2011**, *44*, 101 –110.

(10) Chai, J.-D.; Head-Gordon, M. Systematic optimization of long-range corrected hybrid density functionals. *Phys. Chem. Chem. Phys.* **2008**, *10*, 6615–6620.

(11) Gaussian 09, Revision **E.01**, Gaussian 09, Revision E.01, Frisch, M. J., Trucks, G. W., Schlegel, H. B., Scuseria, G. E., Robb, M. A., Cheeseman, J. R., Scalmani, G., Barone, V., Mennucci, B., Petersson, G. A., Nakatsuji, H., Caricato, M., Li, X., Hratchian, H. P., Izmaylov, A. F., Bloino, J., Zheng, G., Sonnenberg, J. L., Hada, M., Ehara, M., Toyota, K., Fukuda, R., Hasegawa, J., Ishida, M., Nakajima, T., Honda, Y., Kitao, O., Nakai, H., Vreven, T., Montgomery, J. A., Jr., Peralta, J. E., Ogliaro, F., Bearpark, M., Heyd, J. J., Brothers, E., Kudin, K. N., Staroverov, V. N., Kobayashi, R., Normand, J., Raghavachari, K., Rendell, A., Burant, J. C., Iyengar, S. S., Tomasi, J., Cossi, M., Rega, N., Millam, J. M., Klene, M., Knox, J. E., Cross, J. B., Bakken, V., Adamo, C., Jaramillo, J., Gomperts, R., Stratmann, R. E., Yazyev, O., Austin, A. J., Cammi, R., Pomelli, C., Ochterski, J. W., Martin, R. L., Morokuma, K., Zakrzewski, V. G., Voth, G. A., Salvador, P., Dannenberg, J. J., Dapprich, S., Daniels, A. D., Farkas, Ö., Foresman, J. B. Gaussian, Inc., Wallingford CT, **2009**.

(12) (a) Hay, P. J.; Wadt, W. R. Ab initio effective core potentials for molecular calculations. Potentials for the transition metal atoms Sc to Hg. *J. Chem. Phys.* **1985**, *82*, 270–283. (b) Hay, P. J.; Wadt, W. R. Ab initio effective core potentials for molecular calculations. Potentials for K to Au including the outermost core orbitals. *J. Chem. Phys.* **1985**, *82*, 299–310.

(13) Ehlers, A. W.; Biihme, M.; Dapprich, S.; Gobbi, A.; Hijllwarth, A.; Jonas, V.; Kiihler, K. F. ; Stegmann, R.; Veldkamp, A.; Frenking, G. A set of f-polarization functions for pseudo-potential basis sets of the transition metals Sc-Cu, Y-Ag and La-Au. *Chem. Phys. Lett.* **1993**, *208*, 111–114.

(14) Marenich, A. V.; Cramer, C. J.; Truhlar, D. G. Universal Solvation Model Based on Solute Electron Density and on a Continuum Model of the Solvent Defined by the Bulk Dielectric Constant and Atomic Surface Tensions. *J. Phys. Chem. B*, **2009**, *113*, 6378–6396.

(15) NBO 6.0. Glendening, E. D.; Badenhoop, J. K.; Reed, A. E.; Carpenter, J. E.; Bohmann, J. A.; Morales, C. M.; Landis, C. R.; Weinhold, F. (Theoretical Chemistry Institute, University of Wisconsin, Madison, WI, 2013); http://nbo6.chem.wisc.edu/

(16) Bader, R. F. W. Atoms in Molecules: A Quantum Theory; Oxford University Press, 1990.

(17) Lu, T.; Chen, F. *J.* Multiwfn: A multifunctional wavefunction analyzer. *Comput. Chem.*, **2012**, *33*, 580–592.

(18) Pipek, J.; Mezey, P. G. A fast intrinsic localization procedure applicable for ab initio and semiempirical linear combination of atomic orbital wave functions. *J. Chem. Phys.* **1989**, *90*, 4916–4926.

(19) Zou J. MOKIT program, hpps://gitlab.com/jxzou/mokit (accessed Apr 13, 2024).

(20) Bickelhaupt, F. M.; Houk, K. N. Analyzing Reaction Rates with the Distortion/Interaction-Activation Strain Model. *Angew. Chem. Int. Ed.* **2017**, *56*, 10070–10086.

(21) CrysAlisPro Software system, version 1.171.33.51, 2009, Oxford Diffraction Ltd, Oxford, UK.

(22) Dolomanov, O. V.; Bourhis, L. J.; Gildea, R. J.; Howard, J. A. K.; Puschmann, H. OLEX2: a complete structure solution, refinement and analysis program. *J. Appl. Cryst.* **2009**, *42*, 339–341.

(23) Sheldrick, G. M. SHELXT – Integrated space-group and crystal-structure determination. *Acta Cryst.* **2015**, *A71*, 3–8.

(24) Sheldrick, G. M. Crystal structure refinement with *SHELXL*. *Acta Cryst.* **2015**, *C71*, 3–8.
